# Coordination Chemistry of Nucleotides and Antivirally Active Acyclic Nucleoside Phosphonates, including Mechanistic Considerations †

**DOI:** 10.3390/molecules27092625

**Published:** 2022-04-19

**Authors:** Astrid Sigel, Helmut Sigel, Roland K. O. Sigel

**Affiliations:** 1 Department of Chemistry, University of Basel, St. Johannsring 19, CH-4056 Basel, Switzerland; astrid.sigel@unibas.ch; 2 Department of Chemistry, University of Zurich, Winterthurerstrasse 190, CH-8057 Zurich, Switzerland

**Keywords:** acyclic nucleoside phosphonates, antivirals, cell organelles, competing solvent effects, complex stabilities, dephosphorylation, hydrolysis of ATP, intramolecular equilibria, isodesmic model, kinases, mechanistic considerations, metal ion complexes, mixed ligand complexes, nucleic acids, nucleotide analogues, 9-[2-(phosphonomethoxy)ethyl]adenine (PMEA), polarity changes, polymerases, self-association, solvent effects, ternary complexes, triphosphate coordination modes

## Abstract

Considering that practically all reactions that involve nucleotides also involve metal ions, it is evident that the coordination chemistry of nucleotides and their derivatives is an essential corner stone of biological inorganic chemistry. Nucleotides are either directly or indirectly involved in all processes occurring in Nature. It is therefore no surprise that the constituents of nucleotides have been chemically altered—that is, at the nucleobase residue, the sugar moiety, and also at the phosphate group, often with the aim of discovering medically useful compounds. Among such derivatives are acyclic nucleoside phosphonates (ANPs), where the sugar moiety has been replaced by an aliphatic chain (often also containing an ether oxygen atom) and the phosphate group has been replaced by a phosphonate carrying a carbon–phosphorus bond to make the compounds less hydrolysis-sensitive. Several of these ANPs show antiviral activity, and some of them are nowadays used as drugs. The antiviral activity results from the incorporation of the ANPs into the growing nucleic acid chain—i.e., polymerases accept the ANPs as substrates, leading to chain termination because of the missing 3′-hydroxyl group. We have tried in this review to describe the coordination chemistry (mainly) of the adenine nucleotides AMP and ATP and whenever possible to compare it with that of the dianion of 9-[2-(phosphonomethoxy)ethyl]adenine (PMEA^2−^ = adenine(N9)-CH_2_-CH_2_-O-CH_2_-${\mathrm{PO}}_{3}^{2}$) [or its diphosphate (PMEApp^4−^)] as a representative of the ANPs. Why is PMEApp^4−^ a better substrate for polymerases than ATP^4−^? There are three reasons: (i) PMEA^2−^ with its *anti*-like conformation (like AMP^2−^) fits well into the active site of the enzyme. (ii) The phosphonate group has an enhanced metal ion affinity because of its increased basicity. (iii) The ether oxygen forms a 5-membered chelate with the neighboring phosphonate and favors thus coordination at the P_α_ group. Research on ANPs containing a purine residue revealed that the kind and position of the substituent at C2 or C6 has a significant influence on the biological activity. For example, the shift of the (C6)NH_2_ group in PMEA to the C2 position leads to 9-[2-(phosphonomethoxy)ethyl]-2-aminopurine (PME2AP), an isomer with only a moderate antiviral activity. Removal of (C6)NH_2_ favors N7 coordination, e.g., of Cu^2+^, whereas the ether O atom binding of Cu^2+^ in PMEA facilitates N3 coordination via adjacent 5- and 7-membered chelates, giving rise to a Cu(PMEA)_cl/O/N3_ isomer. If the metal ions (M^2+^) are M(α,β)-M(γ)-coordinated at a triphosphate chain, transphosphorylation occurs (kinases, etc.), whereas metal ion binding in a M(α)-M(β,γ)-type fashion is relevant for polymerases. It may be noted that with diphosphorylated PMEA, (PMEApp^4−^), the M(α)-M(β,γ) binding is favored because of the formation of the 5-membered chelate involving the ether O atom (see above). The self-association tendency of purines leads to the formation of dimeric [M_2_(ATP)]_2_(OH)^−^ stacks, which occur in low concentration and where one half of the molecule undergoes the dephosphorylation reaction and the other half stabilizes the structure—i.e., acts as the “enzyme” by bridging the two ATPs. In accord herewith, one may enhance the reaction rate by adding AMP^2−^ to the [Cu_2_(ATP)]_2_(OH)^−^ solution, as this leads to the formation of mixed stacked Cu_3_(ATP)(AMP)(OH)^−^ species, in which AMP^2−^ takes over the structuring role, while the other “half” of the molecule undergoes dephosphorylation. It may be added that Cu_3_(ATP)(PMEA) or better Cu_3_(ATP)(PMEA)(OH)^−^ is even a more reactive species than Cu_3_(ATP)(AMP)(OH)^−^. – The matrix-assisted self-association and its significance for cell organelles with high ATP concentrations is summarized and discussed, as is, e.g., the effect of tryptophanate (Trp^−^), which leads to the formation of intramolecular stacks in M(ATP)(Trp)^3−^ complexes (formation degree about 75%). Furthermore, it is well-known that in the active-site cavities of enzymes the dielectric constant, compared with bulk water, is reduced; therefore, we have summarized and discussed the effect of a change in solvent polarity on the stability and structure of binary and ternary complexes: Opposite effects on charged O sites and neutral N sites are observed, and this leads to interesting insights.


**Table of Contents**
1. Some General Considerations 2. Why Is the Antiviral PMEApp^4−^ a Better Substrate for Nucleic Acid Polymerases than (2’-Deoxy)Adenosine 5′-Triphosphate (dATP^4−^/ATP^4−^)? 3. Modelling the Interactions of Metal Ions with the Ether Oxygen of PMEA^2−^
4. The N3 versus N7 Metal Ion-Binding Mode in Acyclic Nucleoside Phosphonates (ANPs) Containing a Purine Moiety     4.1. The Metal Ion-Coordinating Properties of 9-[2-(Phosphonomethoxy)ethyl]-2-aminopurine (PME2AP)     4.2. The Metal Ion-Coordinating Properties of 9-[2-(Phosphonomethoxy)ethyl]adenine (PMEA)     4.3. The Metal Ion-Coordinating Properties of 9-[2-(Phosphonomethoxy)ethyl]-2,6-diaminopurine (PMEDAP) 5. The Various Inhibiting Substituents in 9-[2-(Phosphonomethoxy)ethyl]-2-amino-6-dimethylaminopurine (PME2A6DMAP) Lead to PME-Like Metal Ion-Binding Properties 6. Mechanistic Considerations on the Metal Ion-Promoted Dephosphorylation of ATP     6.1. Effects of Increasing Amounts of Metal Ions on the Dephosphorylation Rate     6.2. Oligo Formation and the Effect of Self-Association on the Dephosphorylation Rate     6.3. Promotion of ATP Hydrolysis by AMP and PMEA, and Inhibition of the Reactivity by Adenine-Altered AMP Relatives     6.4. Matrix-Assisted Self-Association and Its Significance for Cell Organelles with High ATP Concentrations 7. Solution Structures of Mixed-Ligand Complexes Containing ATP^4−^ and Related Ligands     7.1. Ligands Containing a Ribose Residue with a Phosphate Group     7.2. Acyclic Nucleoside Phosphonates. Cu(arm)(PMEA) as an Example with Intramolecular Stack Formation 8. The Effect of a Change in Solvent Polarity on the Stability and Structure of Binary and Ternary Complexes     8.1. The Dianion of (Phosphonomethoxy)ethane (PME^2−^) as an Example of a Polar O-Ligand. The Properties of Cu(PME) Are Largely as Expected     8.2. Nitrogen Donor Sites Are Especially Sensitive to Polarity Changes of the Solvent     8.3. Competing Solvent Effects on N- versus O-Sites in the Same Ligand and Its Bearing on Complex Stability 9. Discussion 10. Conclusions and Outlook Abbreviations and Definitions References 

## 1. Some General Considerations

**Figure 1:**
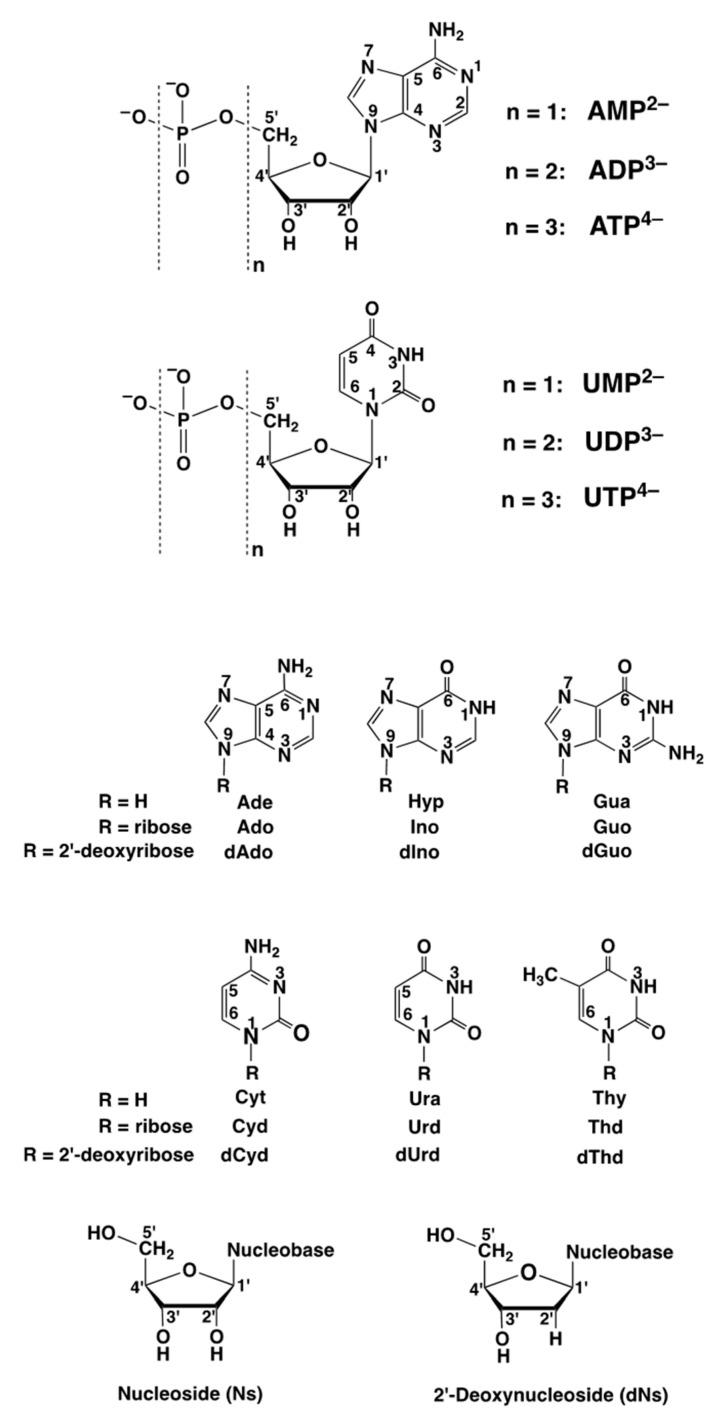
In the *upper part* are shown the chemical structures of the adenosine and uridine 5′-phosphates—namely, adenosine 5′-monophosphate (AMP^2−^; n = 1), adenosine 5′-diphosphate (ADP^3−^; n = 2), and adenosine 5′-triphosphate (ATP^4−^; n = 3), as well as those of uridine 5′-monophosphate (UMP^2−^; n = 1), uridine 5′-diphosphate (UDP^3−^; n = 2), and uridine 5′-triphosphate (UTP^4−^; n = 3). The phosphate groups are named α, β, γ; the γ group being the terminal one. The nucleotides are depictured in their dominating *anti* conformation [4,5,6,7], which means that the adenine residue is pointing away from the ribose plane as is the (C2)O group in the uridine 5′-phosphates. It is obvious that the substitution with other nucleobases will also lead to the *anti* conformation. In the *lower part* of the figure, the structures are shown of the nucleobases (Nb), of the nucleosides (Ns), and of the 2′-deoxynucleosides (dNs). The *Abbreviations* employed (following the order given in the figure) are as follows: Ade = adenine, Ado = adenosine, and dAdo = 2′-deoxyadenosine; Hyp = hypoxanthine, Ino = inosine, and dIno = 2′-deoxyinosine; Gua = guanine, Guo = guanosine, and dGuo = 2′-deoxyguanosine; Cyt = cytosine, Cyd = cytidine, and dCyd = 2′-deoxycytidine; Ura = uracil, Urd = uridine, and dUrd = 2′-deoxyuridine; Thy = thymine; Thd = thymidine, and dThd = 2′-deoxythymidine = 2′-deoxy-5-methyluridine.

Nucleobases (Nb), nucleosides (Ns), and nucleotides, in the form of nucleoside monophosphates (NMP^2−^), nucleoside diphosphates (NDP^3−^), and nucleoside triphosphates (NTP^4−^) (Figure 1; upper part), participate in many biologically relevant reactions, most of them being metal ion-dependent [1,2,3]. Due to the increasing charge and the number of available phosphate groups (denticity), the stability of complexes formed with divalent metal ions increases in the order M(NMP) < M(NDP)^−^ < M(NTP)^2−^. This order is independent of the kind of metal ion considered, though of course, e.g., complexes with Zn^2+^ are considerably more stable than those with Na^+^. In addition, and depending on the circumstances, the nucleobase may also leave its mark (e.g., Section 4 and Section 5), especially if the focus is on selectivity (e.g., Section 2 and Section 7).

The structures of the important nucleobases are depictured in the lower part of Figure 1 [4,5,6,7]. Note, the nucleosides present in ribonucleic acid (RNA) and in deoxyribonucleic acid (DNA) differ: The ribose in RNA carries on OH group in position 2′ of the ribosyl moiety, whereas in the case of DNA at this position only a hydrogen occurs (see Figure 1; bottom part). Moreover, the important nucleobases in RNA are adenine, cytosine, guanine, and uracil, whereas in DNA uracil is replaced by thymine. Next to these five nucleobases there is one more (Figure 1; middle part)—namely, hypoxanthine with its nucleoside, inosine; these compounds (including their phosphates) play important roles in the metabolism of purines [8,9]—i.e., in the derivatives of adenine and guanine.

**Figure 2:**
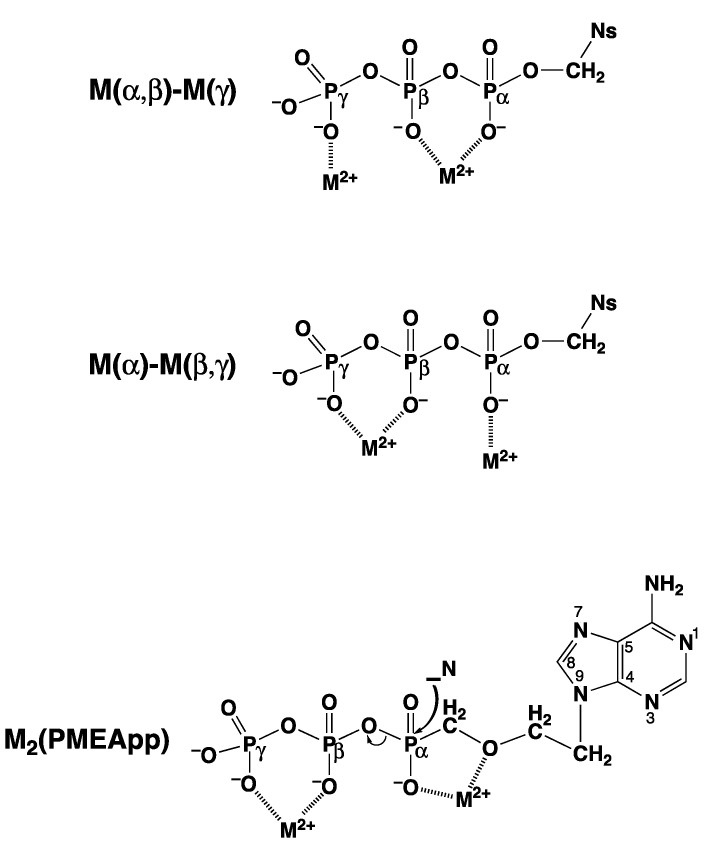
The upper and middle parts of the figure show structures of two M_2_(NTP) complexes, where NTP^4−^ = nucleoside 5′-triphosphate: In one case, a M(α,β)-M(γ) coordination is depictured (*upper part*) indicating the structure relevant for transphosphorylations (kinase, etc.). In the other case, the metal ions are bound in a M(α)-M(β,γ)-type fashion (*middle part*) that is relevant for nucleic acid polymerases, which catalyze the transfer of a nucleotidyl unit. For the latter binding mode, the structure needs to be enforced by the enzyme; this means, the two metal ions need to be anchored [11,12,13,14] to amino acid side chains, often carboxylate groups of aspartate or glutamate residues of the enzyme [15,16,17,18,19]. The (*lower part*) shows a complex formed between two metal ions and diphosphorylated PMEA (9-[2-(phosphonomethoxy)ethyl]adenine)—that is, M_2_(PMEApp). Note, the M(α)-M(β,γ) binding mode, crucial for the polymerase reaction, is favored by the formation of a 5-membered chelate with the ether oxygen of the aliphatic chain (see Section 3). Of course, the adenine residue may be replaced by any other nucleobase moiety and the nucleophile (N) may in addition interact with the M^2+^ at the α-phosphonate group. Altered versions of similar situations are depictured in Refs [20,21,22,23].

The importance of divalent metal ions for the biological transfer of phosphoryl or nucleotidyl groups has long been recognized [10,11,12,13]. It is evident that there are various ways in which a triphosphate chain can coordinate to a metal ion: All three phosphate groups, depending on the size of the metal ion, could possibly directly bind, but also outersphere interactions are possible, etc. In the upper part of Figure 2 [11,14,15,16,17,18,19,20,21,22,23] two structures are indicated that are of relevance with regard to the reactions that are known to occur. The structure at the top shows two metal ions coordinated in such a manner that the terminal γ-phosphate group is activated for transfer [11], thus giving rise to a kinase-type reaction; indeed, such a transphosphorylation mechanism was confirmed for *Escherichia coli* phosphoenolpyruvate carboxykinase by an X-ray structure [24]. Similarly, in the M(α)-M(β,γ) coordinated complex, the break between P_α_ and P_β_ is activated leading to a nucleotidyl transfer—that is, a polymerase-type reaction, as was concluded from solution [11,22,25] as well as solid state studies [15,16,17,18,19,26]. Evidently, the means that favor the one or other activated species need to be discussed (see Section 2 and Section 6).

Of special interest is the structure at the bottom of Figure 2 ([11,14,15,16,17,18,19,20,21,22,23]), where two metal ions are coordinated to diphosphorylated PMEA, (9-[2-(phosphonomethoxy)ethyl]adenine [20,21,22,23]), giving rise to an acyclic ATP analogue. A nucleophilic attack at P_α_ is clearly favored by formation of a 5-membered chelate with the ether oxygen of the aliphatic chain [25]. The effect of a phosphonate P_α_ compared with a phosphate P_α_ needs to be considered in detail (see Section 2).

## 2. Why Is the Antiviral PMEApp^4−^ a Better Substrate for Nucleic Acid Polymerases than (2′-Deoxy)Adenosine 5′-Triphosphate (dATP^4−^/ATP^4−^)?

The advent of the human immunodeficiency viruses (HIV-1 and HIV-2) [27,28,29] spurred research on antivirals. As the virus uses the cellular machinery of the host for its own reproduction (for details see [22,30]), the transcription machinery including nucleotide derivatives of such compounds were also developed and studied in detail. Nucleotides were systematically altered at the nucleobase, at the sugar moiety, and at the phosphate group (e.g., thiophosphate [31,32,33]) with the aim of discovering compounds that inhibit the synthesis of viral DNA or RNA. Among early successful compounds were acyclic nucleoside phosphonates [30], which have the advantage that the phosphorus–carbon bond is not split by dephosphorylation enzymes and that they have no OH group available that allows the continuation of a growing nucleic acid chain—instead, the incorporation of such a derivative into the growing chain leads to its termination [34,35].

9-[2-(Phosphonomethoxy)ethyl]adenine (PMEA) is one of these acyclic nucleoside phosphonates [34], and we will concentrate here on this example. As pointed out already in the preceding Section, this adenine derivative needs to be diphosphorylated to PMEApp^4−^, which is an ATP/dATP analogue, as is indicated in the lower part of Figure 2. In fact, initially PMEApp^4−^ is a better substrate for polymerases than (d)ATP [34] for three main reasons:

- PMEA has an *anti*-like conformation just as AMP.
- The phosphonate group owns an increased basicity and therefore also an enhanced metal ion affinity, and
- formation of a 5-membered chelate involving the ether oxygen favors coordination at the P_α_ group.

These will be discussed below.

**Figure 3:**
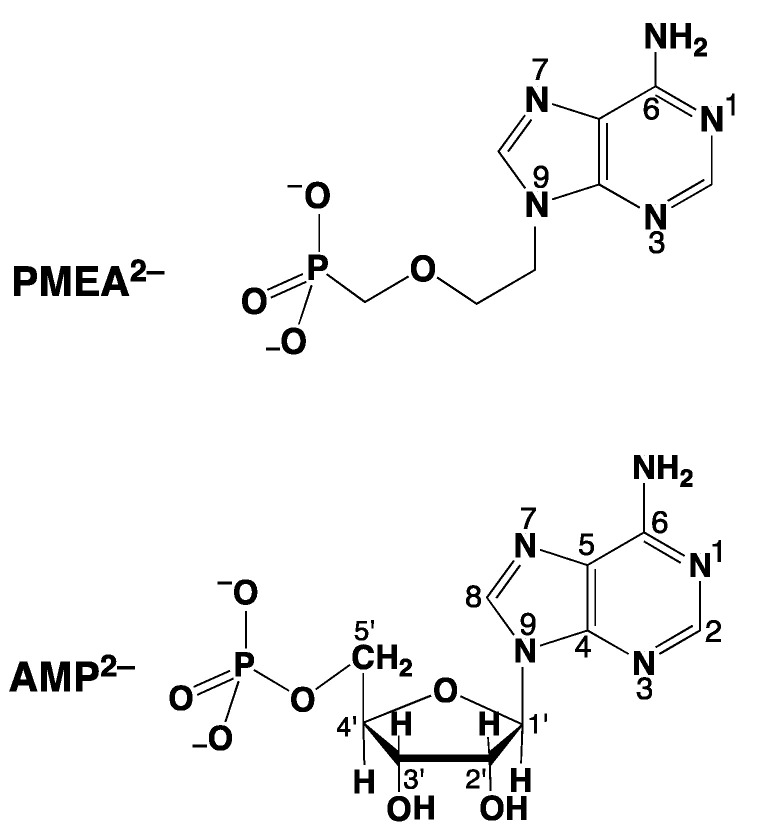
Comparison of the chemical structure of the dianion of 9-[2-(phosphonomethoxy)ethyl]adenine (PMEA^2−^) with the one of adenosine 5′-monophosphate (AMP^2−^). The orientation of PMEA^2−^ in solution [36] and in the solid state [37] resembles the *anti* conformation of AMP^2−^ [4,5,6,38] and thus PMEA^2−^ may be considered as an analogue of AMP^2−^ or 2′-deoxy-AMP^2−^.

In Figure 3, the *anti*-like conformation of PMEA^2−^ [36,37] is shown, and it is expected that this also holds for PMEApp^4−^ ([4,5,6,36,37,38]). Hence, PMEApp^4−^ is further expected to fit into the active site of a polymerase just as well as ATP^4−^ or dATP^4−^.

An oxygen atom is more electronegative than a carbon atom. Therefore, a P–O bond is more polar than a P–C bond, and a phosphonyl group is more basic than a phosphoryl one. This conclusion is confirmed by the data of Table 1 [39,40,41], where the proton affinities (and thus the basicities) of methyl phosphate and methylphosphonate (=P^2−^) are compared [39,40,41,42,43] based on the following two deprotonation equilibria:

(1) $$H_{2}(P)\;⇌{H(P)}^{-}+H^{+}\;\;\;\;\;\;\;\;\;\;\;K_{H_{2}\left(P\right)}^{H}=[H(P{)}^{-}][H^{+}]/[H_{2}(P)]$$

(2) $$H(P{)}^{-}\;⇌P^{2-}+H^{+}\;\;\;\;\;\;\;\;\;\;\;K_{H(P)}^{H}=[H(P{)}^{-}][H^{+}]/[H_{2}(P)]$$

The average difference *Δ* p*K*_a_ = ${pK}_{H(P)}^{H}$ − ${pK}_{H_{2}\left(P\right)}^{H}$ = 5.3 ± 0.2 encompasses the two individual values for CH_3_OPO(OH)_2_ (5.26 ± 0.2) and CH_3_PO(OH)_2_ (5.41 ± 0.03). Indeed, these values are in the expected order [41,42,43], and the differences for phosphates and phosphonates are alike, even though the actual acidity constants differ (Table 1).

An enhanced basicity should give rise to an enhanced metal ion affinity. This postulate can be probed by constructing plots of log $K_{{M(R-\mathrm{PO}}_{3})}^{M}$ (y-axis) versus ${pK}_{{H(R-\mathrm{PO}}_{3})}^{H}$ (x-axis), where ${R-\mathrm{PO}}_{3}^{2-}$ is a phosphate or phosphonate ligand. Indeed, if the residue R does not interfere with metal ion binding (neither in a positive nor negative way), all corresponding data points fit on straight lines, as has been shown for more than 10 metal ions [44,45,46,47]. These lines can be quantified with the straight-line Equation (3)

y = *m* · x + *b* (3)

where x represents the p*K*_a_ value of the phosphate monoester or phosphonate ligand and y the log stability constant of the complex. The slope *m* and the intercept *b* with the y-axis are characteristic for a given metal ion.

We consider now as an example the situation with zinc(II), an important bio-metal ion (Equation (4)):

(4) $$\log\;K_{{\mathrm{Zn}(R-\mathrm{PO}}_{3})}^{\mathrm{Zn}}=(0.345\;\pm \;0.026)\;{pK}_{{H(R-\mathrm{PO}}_{3})}^{H}-(0.017\;\pm \;0.171)$$

This straight-line equation holds in the p*K*_a_ range of about 5 to 8 with an error limit of ±0.060 log units (3σ) [45,46,47].

Application of ${pK}_{{\mathrm{CH}}_{3}{\mathrm{OP}(O)}_{2}\mathrm{OH}}^{H}$ = 6.36 (Table 1) to Equation (4) gives log $K_{{\mathrm{Zn}(\mathrm{CH}}_{3}{\mathrm{OPO}}_{3})}^{\mathrm{Zn}}$ = 2.18 ± 0.06, and application of ${pK}_{{\mathrm{CH}}_{3}{P(O)}_{2}\mathrm{OH}}^{H}$ = 7.51 (Table 1) leads to log $K_{{\mathrm{Zn}(\mathrm{CH}}_{3}{\mathrm{PO}}_{3})}^{\mathrm{Zn}}$ = 2.57 ± 0.06; hence, the phosphonate complex Zn(CH_3_PO_3_) is by 0.39 ± 0.08 log units more stable than the phosphate complex Zn(CH_3_OPO_3_). Or, to express it differently, the Zn^2+^-phosphonate complex is by a factor of 2.5 more stable than the Zn^2+^-phosphate one.

In the structure at the bottom of Figure 2, it is indicated that metal ion coordination is at the P_α_ group—that is, next to the increased basicity of this group—apparently further favored by the formation of a 5-membered chelate involving the ether oxygen. This suspicion will be dealt with in the next Section 3.

## 3. Modelling the Interactions of Metal Ions with the Ether Oxygen of PMEA^2−^

**Figure 4:**
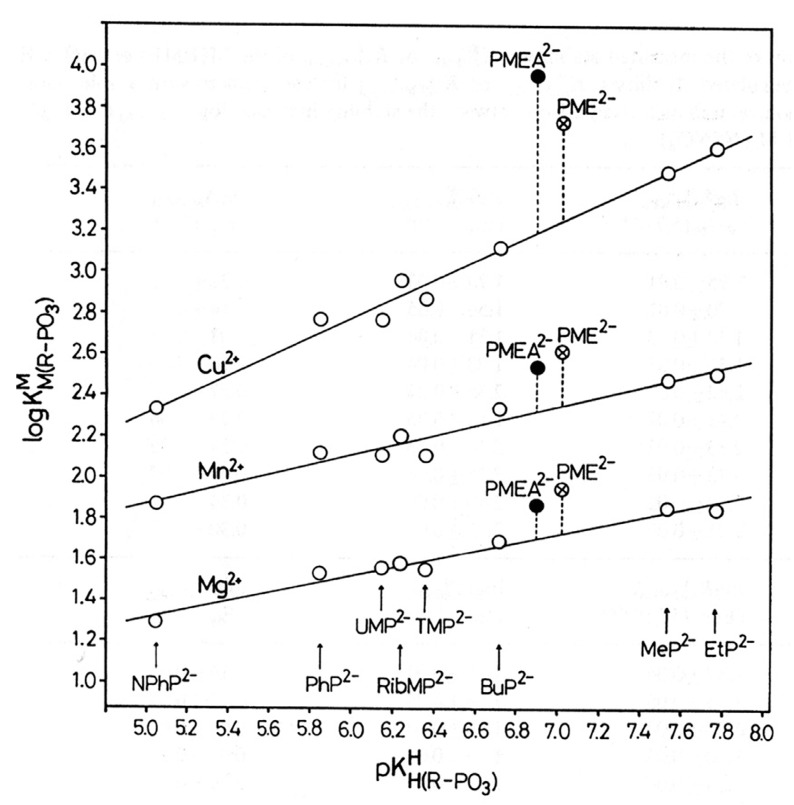
Evidence for enhanced stabilities of some M(PMEA) (●) complexes in comparison with those of the corresponding M(PME) (⊗) species, based on the relationship between log $K_{{M(R-\mathrm{PO}}_{3})}^{M}$ and ${pK}_{{H(R-\mathrm{PO}}_{3})}^{H}\;$ for M(R-PO_3_) complexes of some simple phosphate monoester and phosphonate ligands (${R-\mathrm{PO}}_{3}^{2–}$ ) (◯): 4-nitrophenyl phosphate (NPhP^2−^), phenyl phosphate (PhP^2−^), uridine 5′-monophosphate (UMP^2−^), D-ribose 5-monophosphate (RibMP^2−^), thymidine [= 1-(2′-deoxy-β-D-ribofuranosyl)thymine] 5′-monophosphate (TMP^2−^), *n*-butyl phosphate (BuP^2−^), methanephosphonate (MeP^2−^), and ethanephosphonate (EtP^2−^) (from left to right). The least squares lines (Equation (3)) are drawn through the corresponding 8 data sets (◯) taken from Ref [48] for the phosphate monoesters and from Ref [44] for the phosphonates. The data due to the M^2+^/PMEA (●) and the M^2+^/PME (⊗) systems are from Ref [44]. The vertical broken lines emphasize the stability differences to the reference lines; they equal log *Δ* as defined in Equation (12) for the M(PME) complexes (for the M(PMEA) complexes the analogous formulation holds). All the plotted equilibrium constants refer to aqueous solutions at 25 °C and *I* = 0.1 M (NaNO_3_). Reproduced with permission from our publication in *Coordination Chemistry Reviews* [45]; copyright 1995, Elsevier Science S.A., Lausanne, Switzerland.

To be unequivocal in the evaluations, we need a ligand that offers for metal ion binding only the phosph(on)ate group [48] and the ether oxygen and that should structurally be as close to PMEA^2−^ as possible. These requests are fulfilled by the dianion of ethoxymethanephosphonate, also addressed as (phosphonomethoxy)ethane (=PME^2−^), which is PMEA^2−^ that has lost its adenine residue. As outlined in the preceding Section 2, plots of log $K_{{M(R-\mathrm{PO}}_{3})}^{M}$ (y-axis) versus ${pK}_{{H(R-\mathrm{PO}}_{3})}^{H}$ (x-axis), where R of the ligand ${R-\mathrm{PO}}_{3}^{2-}$ is a residue that does in no way interfere with metal ions, give rise to straight-lines (Equation (3)). Figure 4 shows the straight-line examples for Mg^2+^, Mn^2+^, and Cu^2+^ [44,45].

The data points for the corresponding complexes of PMEA^2−^ and PME^2−^ show that at least in the case of Cu^2+^ the adenine residue participates in metal ion binding because Cu(PMEA) is more stable than Cu(PME) (see Figure 4). More important for the present is the observation that the M(PME) complexes are more stable than is predicted based on the basicity of the phosphonate group; hence, the following intramolecular Equilibrium (5) must be of relevance:



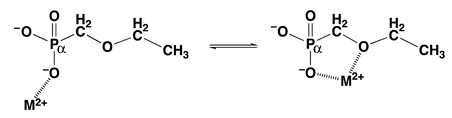

(5)


Its position is determined by the intramolecular, and dimension-less, equilibrium constant *K*_I_ and the ratio of the “closed” (cl) and “open” isomers (Equation (6) where for the present PME^2−^ = PE^2−^):

(6) $$K_{I}=\frac{{\left[M(\mathrm{PE}\right)}_{\mathrm{cl}}]}{{\left[M(\mathrm{PE}\right)}_{\mathrm{op}}]}$$

Consequently, the overall complex formation is described by Equilibrium (7)

(7) $$M^{2+}+{\mathrm{PE}}^{2-}\;⇌\;{M(\mathrm{PE})}_{\mathrm{op}}\;⇌\;{M(\mathrm{PE})}_{\mathrm{cl}}$$

and the experimentally measured stability of the M(PE) complex by Equation (8):

(8) $$K_{M(\mathrm{PE})}^{M}=\frac{{\left([M(\mathrm{PE}\right)}_{\mathrm{op}}{]+[M(\mathrm{PE})}_{\mathrm{cl}}])}{{[M}^{2+}{][\mathrm{PE}}^{2–}]}\;\;\;\;\;=\frac{{\left[M(\mathrm{PE}\right)}_{\mathrm{op}}]}{{[M}^{2+}{][\mathrm{PE}}^{2–}]}+\frac{{\left[M(\mathrm{PE}\right)}_{\mathrm{cl}}]}{{[M}^{2+}{][\mathrm{PE}}^{2–}]}$$

From Equations (6) and (8) follow [45,47,49,50] Equations (9) and (10):

(9) $$K_{M(\mathrm{PE})}^{M}\;=K_{{M(\mathrm{PE})}_{\mathrm{op}}}^{M}+K_{I}\;\cdot \;K_{{M(\mathrm{PE})}_{\mathrm{op}}}^{M}\;=K_{{M(\mathrm{PE})}_{\mathrm{op}}}^{M}(1+K_{I})$$

(10) $$K_{I}=\frac{K_{M(\mathrm{PE})}^{M}}{K_{{M(\mathrm{PE})}_{\mathrm{op}}}^{M}}-1=10^{\log\;\Delta }-1$$

The stability constant of the open isomer (Equation (11))

(11) $$K_{M(\mathrm{PE}{)}_{\mathrm{op}}}^{M}=[M(\mathrm{PE}{)}_{\mathrm{op}}]/([M^{2+}][{\mathrm{PE}}^{2-}])$$

is not directly accessible by experiments, but it may be calculated by applying the acidity constant $K_{H(\mathrm{PE})}^{H}$ (analogue to Equation (2)) to the straight-line equations, as defined by Equation (3). Now with the stability constant of the open isomer available, the stability-constant difference (= stability enhancement; Figure 4) according to Equation (12)

(12) $$\log\;\Delta =\log\;\Delta_{M(\mathrm{PE})}=\log\;K_{M(\mathrm{PE})}^{M}-K_{M(\mathrm{PE}{)}_{\mathrm{op}}}^{M}\;\;=\log\;K_{M(\mathrm{PE}{)}_{\mathrm{exper}}}^{M}-\log\;K_{M(\mathrm{PE}{)}_{\mathrm{calc}}}^{M}$$

can be calculated; this also defines the second term in the above Equation (10). Furthermore, the log *Δ* values correspond to the vertical dotted lines in Figure 4. From Figure 4, it is evident that the stability enhancements (Equation (12)) for the M(PME) and M(PMEA) complexes can differ. To place this conclusion on solid grounds, the mentioned values are compared according to Equation (13), where for the present PMEA = PE:

*Δ* log *Δ*_M/PE/PME_ = log *Δ*_M/PE_ − log *Δ*_M/PME_ (13)

These results are listed in Table 2 (Column 4).

Evidently, with values for *K*_I_ known, the percentages for the closed or chelated species in Equilibrium (5) can be calculated according to Equation (14):

% M(PE)_cl_ = 100 · *K*_I_/(1 + *K*_I_) (14)

Of course, for any calculation of this type, well-defined error limits are compulsory. The corresponding results are summarized in Table 2 [45].

In Columns 2 and 3 of Table 2, the stability enhancements (Equation (12)) for the M(PMEA) and M(PME) complexes are listed [44,45]. These data indicate for all complexes of the various metal ions that they are more stable than expected based on the basicity of the phosphonate group (see also Figure 4). As these values have the same dimension, they can be directly compared, as expressed by Equation (13); the results of these comparisons are listed in Column 4 of Table 2. Interestingly, for the metal ions Mg^2+^, Ca^2+^, Sr^2+^, Ba^2+^, Mn^2+^, Co^2+^, Zn^2+^, and Cd^2+^, the values for *Δ* log *Δ* are zero within the error limits, meaning that the M(PMEA) and M(PME) complexes for a given metal ion show the same stability enhancement and thus the same extent of metal ion–ether oxygen interaction in Equilibrium (5). The corresponding formation degrees (Equation (14)) of the M(PME)_cl_ isomers are listed in the terminal Column of Table 2; the same formation degrees hold within the error limits for the M(PMEA)_cl_ isomers.

The Cu(PMEA) complex is the sole significant exception in Column 4 of Table 2 that shows a further stability enhancement that is beyond that of Cu(PME)—namely, by about 0.3 log unit (see Table 2, Column 4). A corresponding, though much smaller, effect is observed for Ni(PMEA) with *Δ* log *Δ* = 0.11 ± 0.09, which is just at the edge of significance. However, one has to conclude for both instances that the adenine residue must be responsible for this effect [44,45,51]. A M^2+^–N1 interaction cannot be the reason because a phosphonate-coordinated metal ion cannot reach N1 [44,45]. However, interactions appear possible with the other two N atoms; indeed, macrochelate formation with N7 is well-known for many related systems [11,50,51,52,53,54,55,56]. The situation with N3 [44] is more complicated because N3 can only be reached if the metal ion coordinates simultaneously with the ether O atom (see the PMEA^2−^ structure in the upper part of Figure 3), the result being that a 5-membered and a 7-membered chelate are linked to each other [44,45,51]. It appears that the latter isomer involving N3 dominates (for further details see Section 4). Of course, the total amount of chelated species for these two instances is rather large: % Cu(PMEA)_cl/tot_ = 83 ± 3 and % Ni(PMEA)_cl/tot_ = 50 ± 8 [25,44,45].

## 4. The N3 versus N7 Metal Ion-Binding Mode in Acyclic Nucleoside Phosphonates (ANPs) Containing a Purine Moiety

### 4.1. The Metal Ion-Coordinating Properties of 9-[2-(Phosphonomethoxy)ethyl]-2-aminopurine (PME2AP)

**Figure 5:**
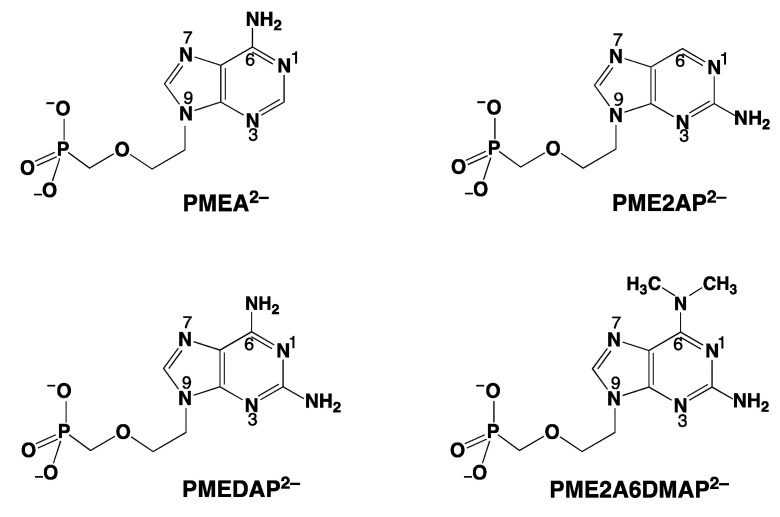
Chemical structures of the dianions of 9-[2-(phosphonomethoxy)ethyl]adenine (PMEA^2−^), 9-[2-(phosphonomethoxy)ethyl]-2-aminopurine (PME2AP^2−^), 9-[2-(phosphonomethoxy)ethyl]-2,6-diaminopurine (PMEDAP^2−^), and 9-[2-(phosphonomethoxy)ethyl]-2-amino-6-dimethylaminopurine (PME2A6DMAP^2−^). These compounds are abbreviated as PE^2−^. For the solution structure of PMEA^2−^, see legend of Figure 3. Replacement of the adenine residue in PMEA^2−^ by a cytosine residue (Figure 1) gives PMEC^2−^ = 1-[2-(phosphonomethoxy)ethyl]cytosine.

Research on acyclic nucleoside phosphonates (ANPs) containing a purine residue revealed that the kind and the position of the substituent (at C2 or C6) has a significant influence on the biological activity. For example, the shift of the (C6)NH_2_ group in PMEA to the C2 position (Figure 5) leads to an ANP isomer, i.e., PME2AP, which shows only a moderate antiviral activity compared with that of PMEA [57].

Of course, the removal of the (C6)NH_2_ substituent removes the steric effect of this group on N7, and consequently, macrochelate formation involving N7 is strongly enhanced in M(PME2AP) species [43,58], whereas no metal ion interaction with N3 could be discovered. This is because the NH_2_ substituent at C2 inhibits metal ion binding to N3. This observation supports the hypothesis that the C6 substituent plays an important role for the biological activity.

**Figure 6:**
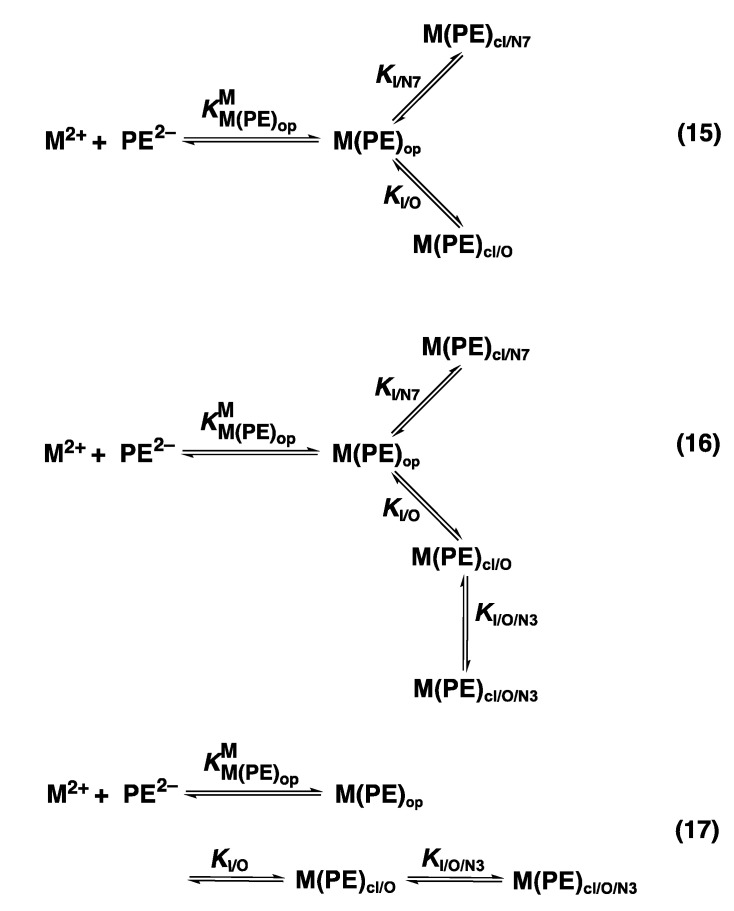
Equilibrium schemes involving various N sites in metal ion complexes of acyclic nucleoside phosphonates containing a purine moiety.

For the M(PME2AP) complexes, the stability increases between 0.13 and 1.06 log units (=log *Δ*_M/PME2AP_) [43]. For the complexes of Mg^2+^, Ca^2+^, (Sr^2+^, Ba^2+^ were not studied), and Mn^2+^, only Equilibrium (5) is responsible for the increased complex stabilities. However, for the complexes of Co^2+^, Ni^2+^, Cu^2+^, Zn^2+^, and Cd^2+^, macrochelate formation is of relevance as well [43], which means that the Equilibrium Scheme (15) in Figure 6 describes the situation.

The formation degrees of the two relevant and chelated species M(PME2AP)_cl/O_ and M(PME2AP)_cl/N7_, plus the “open” species M(PME2AP)_op_ are summarized in Table 3. The larger formation degree of Ni(PME2AP)_cl/N7_ compared with Cu(PME2AP)_cl/N7_ corresponds to the situation with the M(AMP) complexes [50] and can be explained by statistical considerations (see Section 9) based on the different geometries of the coordination spheres of Ni^2+^ and Cu^2+^ [59].

### 4.2. The Metal Ion-Coordinating Properties of 9-[2-(Phosphonomethoxy)ethyl]adenine (PMEA)

The best known ANP of the compounds shown in Figure 5 is PMEA, which exhibits various biological activities [59,60] and which in the form of its bis(pivaloyloxymethyl)ester [22] is used as an oral prodrug (adefovir dipivoxil) already for many years [59]. This neutral prodrug passes membranes more easily than PMEA^2−^, which is released inside the cell and then diphosphorylated giving PMEApp^4−^ as the biologically active dATP^4−^/ATP^4−^ analogue (Section 2).

**Figure 7:**
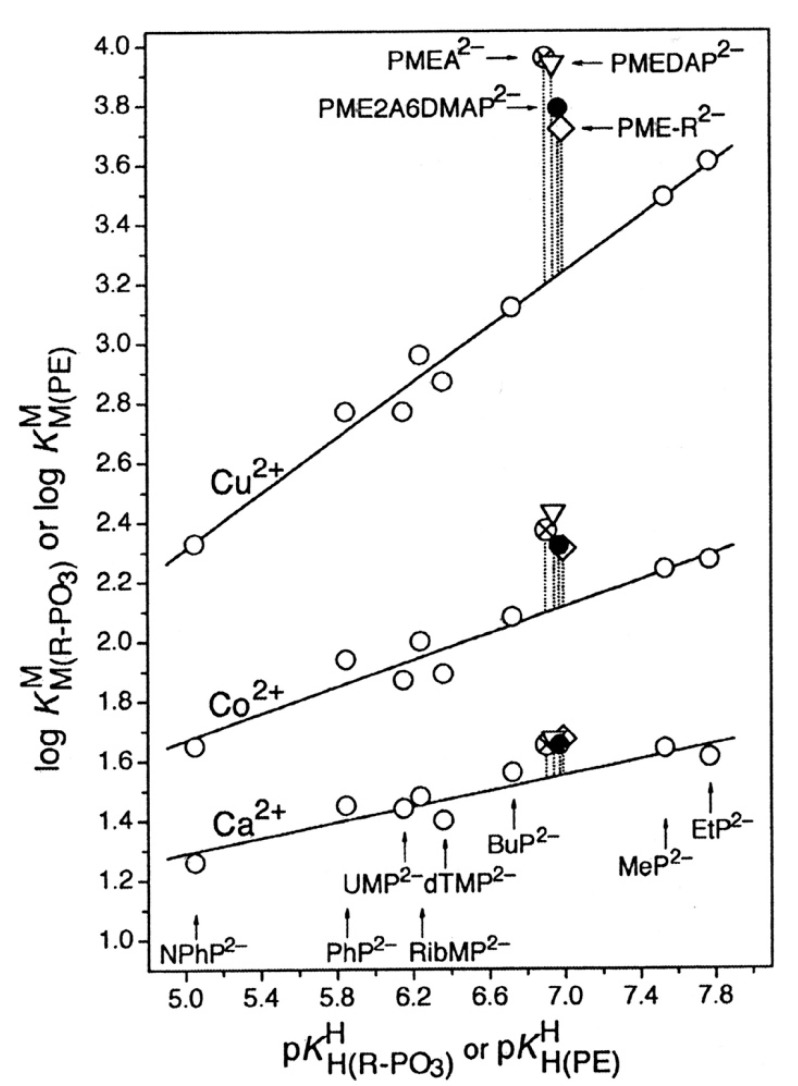
Evidence for enhanced stabilities of some M(PME2A6DMAP) (●) complexes in comparison with those of the corresponding M(PMEA) (⊗, M(PMEDAP) (▽), and M(PME-R) (◇) species, based on the relationship between log $K_{{M(R-\mathrm{PO}}_{3})}^{M}$ and ${pK}_{{H(R-\mathrm{PO}}_{3})}^{H}\;$ for M(R-PO_3_) complexes of some simple phosphate monoester and phosphonate ligands (${R-\mathrm{PO}}_{3}^{2–}$) (◯) (see legend to Figure 4). The points due to the equilibrium constants for the M^2+^/PME2A6DMAP systems (●) are based on the values listed in Tables 1 and 2 of Ref [61]; those for the M^2+^/PMEA systems (⊗) are from Ref [44], for the M^2+^/PMEDAP systems (▽) from Ref [62], and those for the M^2+^/PME-R systems (◇) are based on ${pK}_{H(\mathrm{PME}-R)}^{H}$ = 6.99 ± 0.04 (average of the p*K*_a_ values for H(PME)^−^ (7.02) and H(PMEC)^−^ (6.95) [63]) and the stability enhancements listed in Ref [63]. The vertical broken lines emphasize the stability differences to the reference lines; they equal log *Δ*_M/PE_, as defined in Equation (12) for the M(PE) complexes in general. All the plotted equilibrium constants refer to aqueous solutions at 25 °C and *I* = 0.1 M (NaNO_3_). Reproduced with permission from our publication in the *Canadian Journal of Chemistry* [61]; copyright 2014, NRC Research Press.

For most of the studied M(PMEA) complexes, it is Equilibrium (5) that explains all observed properties (Table 2; Section 3). The significant exception is Cu(PMEA), which is about 0.3 log unit more stable beyond the ether O–metal ion interaction (Section 3). This is also seen in Figure 7 [44,61,62,63] if the stabilities of the Cu(PMEA) and Cu(PME-R) are compared; this extra stability must be attributed to a metal ion interaction with the adenine residue.

Based on steric and chemical considerations [45,64], it was concluded that the Equilibrium Scheme (17) (Figure 6) involving Cu(PMEA)_cl/O/N3_ is the relevant one and not Equilibrium Scheme (15) with Cu(PMEA)_cl/N7_. This conclusion was confirmed by ^1^H NMR line-broadening experiments [65]; hence, the (C6)NH_2_ substituent inhibits Cu^2+^ coordination at N7 and the ether O atom-binding of Cu^2+^ facilitates N3 coordination via adjacent 5- and 7-membered chelate rings.

Later studies with 9-(4-phosphonobutyl)adenine (=3′-deoxa-PMEA^2−^ = dPMEA^2−^) [66] provided evidence for the formation of in total four isomers—that is, in addition to those appearing in the Equilibrium Scheme (17) (Figure 6), also a macrochelated species involving N7, Cu(PMEA)_cl/N7_ may form in traces, and the Equilibrium Scheme (16) is valid. The calculated percentages (in parentheses) [66] for the four isomers are Cu(PMEA)_op_ (17 ± 3%), Cu(PMEA)_cl/O_ (34 ± 10%), Cu(PMEA)_cl/N7_ (7.7 ± 5.3%), and Cu(PMEA)_cl/O/N3_ (41 ± 12%). These results are based on the assumption that the butyl chain of dPMEA^2−^ and the one of PMEA containing the ether oxygen in their formation of the macrochelate with N7 behave alike; there is, however, a small caveat here: The macrochelate with dPMEA^2−^ could be somewhat stabilized by a hydrophobic interaction between the butyl chain and the imidazole ring of the adenine residue; then the given estimation of 7.7 ± 5.3% for Cu(PMEA)_cl/N7_ would be somewhat too large.

### 4.3. The Metal Ion-Coordinating Properties of 9-[2-(Phosphonomethoxy)ethyl]-2,6-diaminopurine (PMEDAP)

We have already noted that PMEA (Figure 5) has antiviral properties and that those of PME2AP are smaller. The related ANP, PMEDAP (Figure 5), is also potentially a useful antiviral compound [34,67,68]. However, PMEDAP has a more pronounced cytostatic effect than PMEA; it affects human leukemia cell lines [69] and inhibits the cellular DNA polymerase δ [70].

PMEDAP is an interesting molecule with an amino group each at C2 and C6. In Figure 7, it is seen that its Cu^2+^ complex has about the same stability as Cu(PMEA) and that both complexes are more stable than Cu(PME-R) (R = non-interfering residue). This proves that the nucleobase residues must be involved in Cu^2+^ binding. Considering that the (C2)NH_2_ group inhibits metal ion binding at N3, this observation seems surprising because N3, like with PMEA (Section 4.2), cannot be the cause. However, due to the further NH_2_ group in PMEDAP, compared with PMEA, at the adenine residue the basicity of the 2,6-diaminopurine moiety is by *Δ* p*K*_a_ of about 0.7 more basic than the adenine residue [62]. This basicity increase enhances the Cu^2+^ affinity for N7 so much that the steric inhibiting effect of (C6)NH_2_ on N7 is compensated. To summarize, Cu(PMEA) has an increased complex stability due to an N3 coordination and Cu(PMEDAP) due to an involvement of N7 in macrochelate formation. These different binding sites in the seemingly similar complexes are confirmed by ^1^H NMR line-broadening experiments [62].

In Table 4 in Columns 2 and 3, the stability enhancements (Equation (12)) for the M(PMEDAP) and M(PME-R) complexes are listed, and in Column 4, these stability enhancements are compared according to Equation (13). This comparison shows that for the M(PMEDAP) complexes of Mg^2+^, Ca^2+^, Sr^2+^, Ba^2+^, Mn^2+^, (Zn^2+^), and Cd^2+^, only Equilibrium (5) is of relevance; hence, the nucleobase moiety does not participate in these instances in metal ion binding. This is different for the complexes of Co^2+^, Ni^2+^, and Cu^2+^, where the 2,6-diaminopurine moiety is involved in metal ion binding (see Section 4.2). That apparently the *Δ* log *Δ* value for Ni(PMEDAP) is a bit larger than that for Cu(PMEDAP) (Table 4, Column 4) corresponds to the observation made (vide supra) with the corresponding M(PME2AP) complexes (Section 4.1), as well as with those for M(AMP), and can be explained, as said, by statistical considerations based on the different geometries of the coordination spheres of Ni^2+^ and Cu^2+^ [50] (see Section 9).

From Column 6 in Table 4, it follows that the formation degrees of the 5-membered chelates, only Equilibrium (5) being of relevance, varies for the six metal ions in question between about 15% (Sr^2+^) and 50% (Cd^2+^). In the case of Co^2+^, Ni^2+^, and Cu^2+^ complexes, as discussed, the macrochelate formed by the phosphonate-coordinated metal ions with N7 is of relevance. The formation degrees of these macrochelates, M(PMEDAP)_cl/N7_, are for Co^2+^, Ni^2+^, and Cu^2+^ 26 ± 14%, 54 ± 10%, and 43 ± 11%, respectively [62].

Interestingly, 9-[2-(phosphonomethoxy)ethyl]-2-amino-6-dimethylaminopurine (PME2A6DMAP) shows similar therapeutic effects as the parent PMEDAP (Figure 5) [60,71], although it exerts even a stronger cytostatic activity in mouse leukemia L1210 cells or in human cervix carcinoma [60]. In the indicated context of the biological activity, it is amazing to note that the metal ion-coordinating properties between these two ANPs differ considerably. Those for PMEDAP were discussed in this Section 4.3; in the next Section 5, those for PME2A6DMAP (Figure 5) will follow.

## 5. The Various Inhibiting Substituents in 9-[2-(Phosphonomethoxy)ethyl]-2-amino-6-dimethylaminopurine (PME2A6DMAP) Lead to PME-Like Metal Ion-Binding Properties

As far as the proton affinity of the N1 site is concerned, we have seen that the introduction of a second amino group into the adenine moiety makes the N1 site by *Δ* p*K*_a_ ca. 0.7 more basic (Section 4.3). A similar situation is observed now for PMEA and PME2A6DMAP, with an enhanced proton affinity of N1 by *Δ* p*K*_a_ ca. 0.9 (${pK}_{H_{2}\left(\mathrm{PMEA}\right)}^{H}$ = 4.16 ± 0.02 and ${pK}_{H_{2}\left(\mathrm{PME}2\mathrm{ADMAP}\right)}^{H}$ = 5.09 ± 0.02 [61]). In contrast, the basicity of the phosphonate group is very similar for the two ANPs [61] (see also the ${pK}_{H(\mathrm{PE})}^{H}$ values in Figure 7). However, this increase in basicity of the nucleic base moiety has no influence on the stability of the Cu^2+^ complexes of PME2A6DMAP^2−^, in contrast with that of PMEDAP^2−^ (Figure 7): The stabilities of Cu(PMEA) and Cu(PMEDAP) are enhanced and very similar, whereas the stability of Cu(PME2A6DMAP) is close to the nucleic base-free Cu(PME-R) complex.

The reason lies in the structure of the M(PME2A6DMAP) complex. The ligand PME2A6DMAP^2−^ offers in principle four metal ion binding sites, N1 not being accessible [44,45] (Section 3), leaving N3, N7, the ether oxygen of the –CH_2_–CH_2_–O–CH_2_–${\mathrm{PO}}_{3}^{2–}$ chain, and the phosphonate group. From Figure 7, it follows that the phosphonate group is the primary metal ion-binding site, and therefore, the data need to be evaluated first for the occurrence of Equilibrium (5). For this reason, we have collected in Column 2 of Table 5 [61] the stability enhancements for log *Δ*_M(PME2A6DMAP)_ = log *Δ*_M(PE)_ (Equation (12)). These data are compared according to Equation (13) with the log *Δ*_M(PME-R)_ values (Table 5, Column 3), which contain solely Equilibrium (5) as a source for an increased complex stability. It is revealing to see that all the values for *Δ* log *Δ* (Equation (13)) in Column 4 of Table 5 are zero within the error limits. From this result for *Δ* log *Δ*, it follows that the nucleobase of PME2A6DMAP^2−^ does not participate in metal ion binding but that the coordination of the ether O atom is solely responsible for the increased complex stability.

Application of log *Δ* = log *Δ*_M(PE)_ = log *Δ*_M(PME2A6DMAP)_ to Equation (10) provides the intramolecular equilibrium constant *K*_I_ = *K*_I/PE_ for Equilibrium (5) (Table 5, Column 5), and from this the formation degree of the M(PME2A6DMAP)_cl/O_ species follows with Equation (14) (Table 5, Column 6). This formation degree varies between about 15% to 70%, depending on the metal ion involved in Equilibrium (5). As one would expect, these formation degrees are within their error limits identical with those listed in the terminating Column of Table 5 for the % M(PME-R)_cl/O_ species. In other words, PME2A6DMAP^2−^ coordinates to metal ions in a PME-like manner, as seen in Equilibrium (5). However, this does not exclude, e.g., stacking interactions [72] between the purine residue of PME2A6DMAP^2−^ in a protein or nucleic acid.

## 6. Mechanistic Considerations on the Metal Ion-Promoted Dephosphorylation of ATP

### 6.1. Effects of Increasing Amounts of Metal Ions on the Dephosphorylation Rate

The rate of hydrolysis of ATP [10] and other NTPs [14,73] increases with decreasing pH—i.e., with an increasing proton concentration [14]. This is confirmed in a quantitative manner by the following considerations: The p*K*_a_ values for the deprotonation of H_2_(ATP)^2−^ are ${pK}_{H(\mathrm{ATP})}^{H}$ = 6.47 ± 0.01 and ${pK}_{H_{2}\left(\mathrm{ATP}\right)}^{H}$ = 4.00 ± 0.01 (25 °C; aqueous solution; *I* = 0.1 M, NaNO_3_ or NaClO_4_) [74]—that is, for the deprotonation of the terminal γ-phosphate group and the (N1)H^+^ site, respectively. The initial rate, v_0_, of the dephosphorylation reaction of ATP in 10^−3^ M aqueous solution at 50 °C and pH_0_ ca. 9 amounts to v_0_ = 1.5 × 10^−10^ M s^−1^; at pH_0_ 5.5 one obtains v_0_ = 10 × 10^−10^ M s^−1^. This means, roughly speaking, at 50 °C and *I* = 0.1 M (Na^+^) H(ATP)^3−^ hydrolyzes by a factor of about 7 faster than ATP^4−^. The addition of one equivalent of Cu^2+^ to the 10^−3^ M ATP solution at 50 °C and pH_0_ 5.5 has significant consequences because now v_0_ = 25 × 10^−9^ M s^−1^; in other words, the dephosphorylation rate is increased by a factor of about 25.

**Figure 8:**
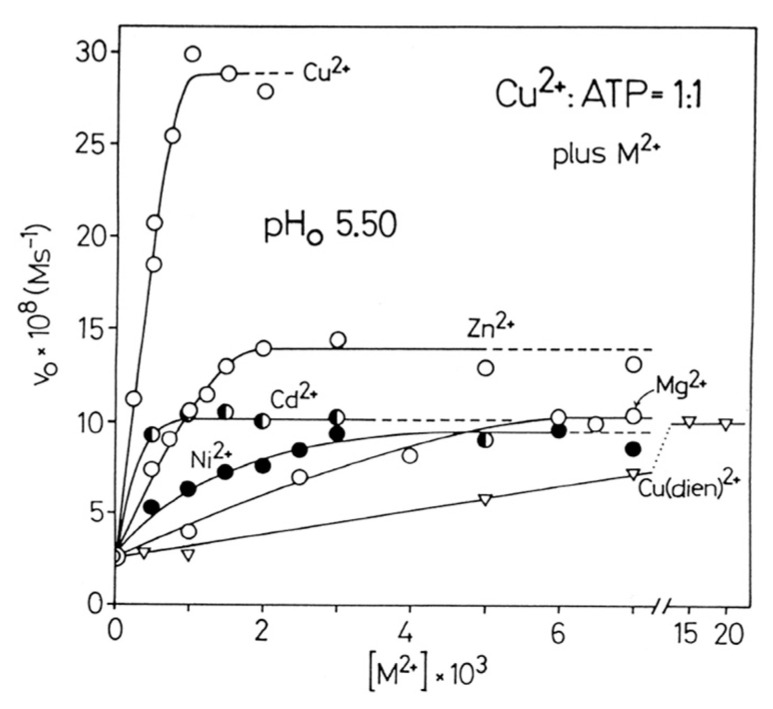
Dependence of the initial rate, v_0_ = d[PO_4_]/dt (M s^−1^), of the Cu^2+^-promoted dephosphorylation of ATP ([Cu^2+^]_tot_ = [ATP]_tot_ = 10^−3^ M) in aqueous solution on the addition of further divalent metal ions (empty circles, half-filled circles, full circles) or the Cu^2+^ 1:1 complex with diethylenetriamine (= dien = 1,4,7-triazaheptane (▽)) at pH_0_ 5.50; *I* = 0.1 M, NaClO_4_; 50 °C. The broken line portions indicate uncertainty due to precipitation. The figure is reproduced with permission from our publication in *Inorganica Chimica Acta* [55]; copyright 1992, Elsevier Sequoia (see also [14]).

The mentioned observations stipulated the experiments shown in Figure 8, where the initial rate v_0_ at pH_0_ 5.5 is plotted for the dephosphorylation reaction in dependence on the concentration of a second metal ion. In the case of Cu^2+^ as the second metal ion, the effect is most pronounced (Figure 8) because with one more equivalent of Cu^2+^ the system reaches already its maximum rate. The reason is that the Cu_2_(ATP) complex is rather stable [10] and that the second Cu^2+^ is present at pH_0_ 5.5 as Cu(OH)^+^, and this allows an intramolecular OH^−^ attack at the terminal γ-phosphate group. This type of reactivity involving OH^−^ applies to some extent also for Zn^2+^, which is an extremely versatile metal ion [75,76].

However, Mg^2+^, Ni^2+^, and Cd^2+^ reach as the second metal ion and as promoters of the dephosphorylation reaction all a limiting v_0_ value of about 10·10^−8^ M s^−1^—that is, compared with the Cu^2+^:ATP = 1:1 system (v_0_ = 2.5 × 10^−8^ M s^−1^), the dephosphorylation rate is enhanced by a factor of about four. Interestingly, such a second metal ion is of relevance also for kinases [77,78,79]. Note, the only difference between the mentioned three metal ions is that the maximum rate of 10 × 10^−8^ M s^−1^ is reached at different concentrations; in other words, the stabilities of these ATP^4−^ complexes decrease in the order Cd^2+^ > Ni^2+^ > Mg^2+^ [74], and the reaction proceeds with H_2_O as the nucleophile.

The situation with Cu(dien)^2+^ as promoter is most interesting because in Cu(dien) only a single equatorial binding site at Cu^2+^ is left. Despite this handicap, with a 15-fold excess of Cu(dien) over Cu(ATP)^2−^, the same maximum rate is obtained as with Mg^2+^, Ni^2+^, and Cd^2+^. Hence, generally speaking, the reactive complex has the composition M[Cu(ATP)]. This is in accord with the observations that for a given metal ion the reactive species has the composition M_2_(ATP), as is confirmed [10,14,80] for several metal ions at various pH values by Job’s plots [81]. In this context, one may mention that polymerases are known (for DNA [25,82,83,84] and RNA [2,25,83,84,85]) that contain an intrinsic divalent metal ion, often Zn^2+^ [82], but in order to be active with a NTP^4−^ substrate, also an extrinsic second divalent metal ion, often Mg^2+^ (or Mn^2+^), is needed [26,84,86,87].

### 6.2. Oligo Formation and the Effect of Self-Association on the Dephosphorylation Rate

Purine nucleotide residues, as they occur in DNA or RNA, form intramolecular stacks and determine in this way the structure of these macromolecules. Such π stacks can also occur between simple purine units [88] leading to associates [89,90,91,92], and their formation is reflected in the ^1^H NMR spectrum that changes with increasing nucleotide concentration. Plots of the changes in dependence on concentration can be successfully interpreted with the isodesmic model of non-cooperative self-association [89]; i.e., it is assumed, e.g., for an adenine derivative (A) that for Equilibrium (18) the association constants for the various steps are all equal (Equation (19)):

(18) $$(A{)}_{n}+A\;⇌\;{\left(A\right)}_{n+1}$$

*K* = [(A)_n+1_]/[(A)_n_][A] (19)

In Table 6, the association constants for adenosine (Ado) and several of its derivatives are listed [89,93]. The comparison of the constants for adenosine and AMP^2−^ shows that the negative charge leads to repulsion and thus inhibits the association; indeed, the constants decrease further for ADP^3−^ and ATP^4−^. However, for Mg(ATP)^2−^, the association increases (*K* = 4.0 ± 0.5 M^−1^) beyond that of AMP^2−^ (*K* = 2.1 ± 0.4 M^−1^), despite the equality of the charges. This indicates that Mg^2+^ does not only compensate part of the negative charge but that this metal ion links in addition the phosphate residues of different ATPs together and that this stabilizes the oligomers further [89].

Yet, for Zn(ATP)^2−^ and Cd(ATP)^2−^, the constants are much larger than those for Mg(ATP)^2−^ (Table 6), indicating that interactions with the adenine residue must now also occur. Indeed, the chemical shifts for (C8)H indicate that at low concentrations a downfield shift occurs, whereas all other shifts are upfield [90]; this suggests the preferred formation of Zn^2+^/N7 bridged ${\left[\mathrm{Zn}(\mathrm{ATP})\right]}_{2}^{4–}$ dimers, which then associate further in an unbridged manner [89]. The analogous observation is made with Cd^2+^/ATP^4−^. For M^2+^ = Zn^2+^ or Cd^2+^, one may write the following two equilibria:

(20) $$2\;M{\left(\mathrm{ATP}\right)}^{2-}\;⇌{\left[M(\mathrm{ATP})\right]}_{2}^{4–}\;\;\;\;\;\;\;\;\;\;\;\;\;\;K_{D}^{*}=[{\left[M(\mathrm{ATP})\right]}_{2}^{4–}]/[M{\left(\mathrm{ATP}\right)}^{2-}{]}^{2}$$

(21) $${\left[M(\mathrm{ATP})\right]}_{2}^{4–}+M_{n}{\left(\mathrm{ATP}\right)}_{n}^{2n–}⇌\;M_{2+n}{\left(\mathrm{ATP}\right)}_{2+n}^{(4+2n)–}\;\;\;\;\;\;\;\;\;\;\;\;\;\;K_{\mathrm{st}}=[M_{2+n}{\left(\mathrm{ATP}\right)}_{2+n}^{(4+2n)–}]/[{\left[M(\mathrm{ATP})\right]}_{2}^{4–}]{{[M}_{n}\left(\mathrm{ATP})\right]}_{n}^{2n–}]$$

**Figure 9:**
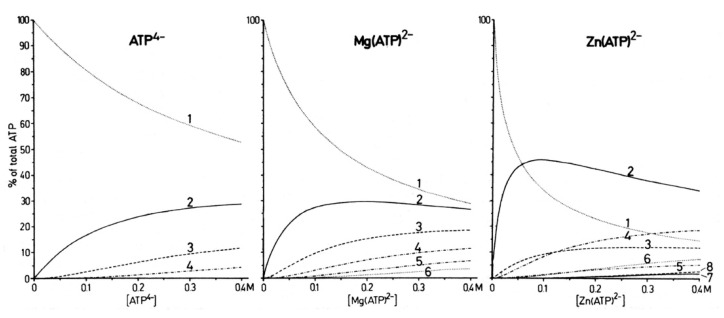
Variation in the proportions of ATP present in the monomer (1), dimer (2), trimer (3), …, and octamer (8) in D_2_O solutions in dependence on the total concentration of ATP^4−^ (*K* = 1.3 M^−1^), Mg(ATP)^2−^ (*K* = 4.0 M^−1^), and Zn(ATP)^2−^ ($K_{D}^{*}$ = 20 M^−1^ and *K*_st_ = 4 M^−1^) (see text in Section 6.2 and Equations (18)–(20)) (27 °C, *I* = 0.1 to ~2 M (NaNO_3_) in D_2_O). This figure is reproduced with permission from our publication in the *Journal of the American Chemical Society* [89]; copyright 1981, American Chemical Society (see also [53]).

In Figure 9, we plotted the formation degrees of monomeric to octameric species in the ATP concentration range 0 to 0.4 M. The preferred formation of dimers in the Zn^2+^/ATP = 1:1 system is evident. These data allow definition of the concentrations one should use if one wants to describe the properties of monomers. For example, in 1 mM solutions of Mg(ATP)^2−^, about 99% are present in the monomeric form, whereas under the same conditions for Zn(ATP)^2−^, only about 96% are monomers [53]. It is thus advisable to work with [ATP] < 1 mM in studies aimed for the properties of monomers.

That for the dephosphorylation reaction of ATP in the presence of the mentioned metal ions, Ni^2+^, Cd^2+^, Cu^2+^, and Zn^2+^, the formation of dimeric species for reaching the reactive state is important (in the presence of one or two equivalents of M^2+^), follows from plots of log v_0_ versus log [ATP]_tot_; ATP being present with one or two equivalents of metal ions. These plots show slopes of two for Cd^2+^, e.g., at pH_0_ 7.2, 8.2, and 9.0 [80], for Cu^2+^ at pH_0_ 5.5 and 6.7, for Ni^2+^ at pH_0_ 8.0, and for Zn^2+^ at pH_0_ 7.2 [10], meaning that the reactive M(ATP)^2−^ or M_2_(ATP) complex is a dimer. The latter species is the more reactive one, as is also confirmed by Job’s plots (Section 6.1); thus [M_2_(ATP)]_2_ or [M_2_(ATP)]_2_OH^−^, depending on the metal ion involved, is the most reactive complex.

**Figure 10:**
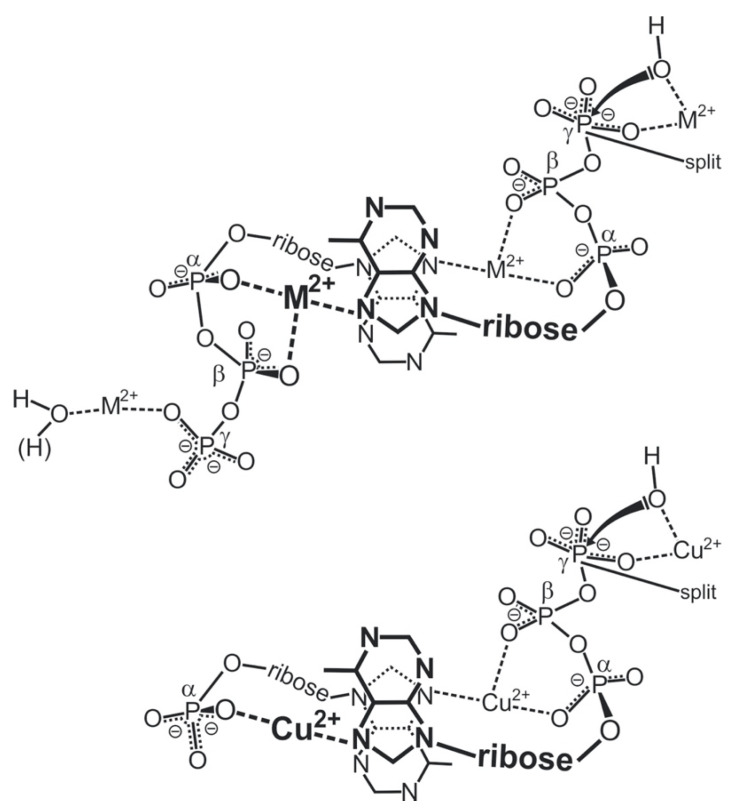
TOP: Proposed structure of the reactive [M_2_(ATP)]_2_(OH)^−^ dimer, which occurs in low concentration during the metal ion-promoted dephosphorylation of ATP. The intramolecular attack of OH^−^ is indicated on the right-hand side, while the left-hand side is ready to transfer also into the reactive state by deprotonation of the coordinated water molecule or to undergo an intramolecular water attack, corresponding to the dimeric [M_2_(ATP)]_2_ species. BOTTOM: Probable structure of the reactive Cu_3_(ATP)(AMP)(OH)^−^ species. The intramolecular attack of OH^−^ is indicated on the right-hand side, while the left-hand side shows the metal ion bridge stabilizing the purine stack by M^2+^ coordination to the phosphate group of AMP^2−^ and to N7 of ATP^4−^ [14,80,94]. The structures are adapted from Ref [80].

The tentative structure of such a dimer is shown in the upper part of Figure 10 [14,80,94]. It is evident that one of the two ATP molecules (left part) is used to organize with a metal ion (M^2+^) a bridge to N7 and to stabilize the stack, whereas the other ATP is in the reactive state for OH^−^ or H_2_O attack and thus for the release of the terminal γ-phosphate group. One could conclude that ATP is its own “enzyme” [55]. Indeed, this view is confirmed in the next section.

### 6.3. Promotion of ATP Hydrolysis by AMP and PMEA, and Inhibition of the Reactivity by Adenine-Altered AMP Relatives

**Figure 11:**
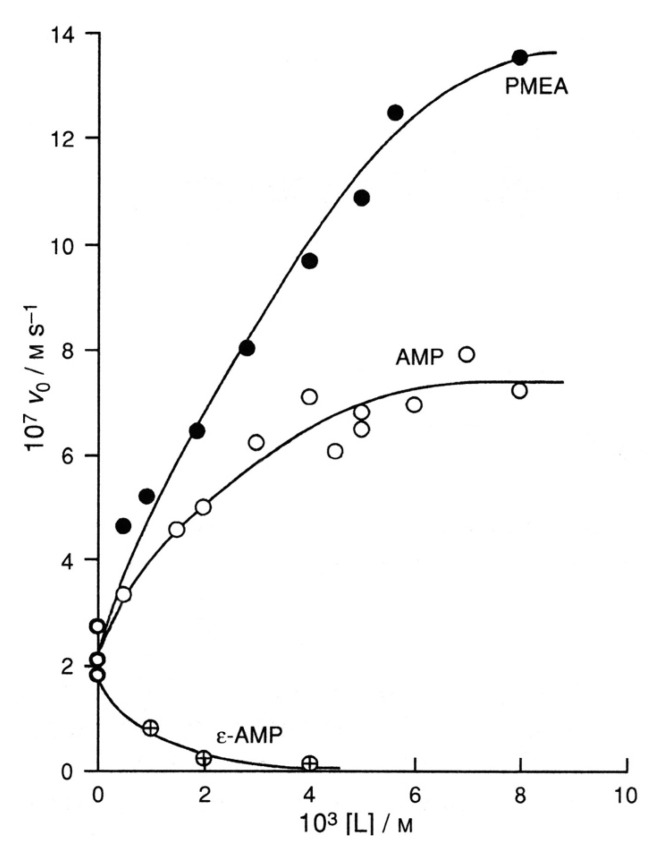
Influence of AMP (◯) and PMEA (●) as well as of ε-AMP (Figure 12) on the initial rate v_0_ (M s^−1^) of the dephosphorylation of the Cu^2+^/ATP 2:1 system ([Cu^2+^]_tot_ = 2 × 10^−3^ M and [ATP]_tot_ = 10^−3^ M) in aqueous solution at pH 6.70 (*I* = 0.1 M, NaClO_4_; 50 °C). This figure is adapted with permission from Figure 3 of our publication in *Chem. Commun.* [94]; copyright 1998, The Royal Society of Chemistry.

The dimer shown in the upper part of Figure 10 consists of two M_2_(ATP) units. The left part of this dimer is the structuring part, and “removing” the β,γ-phosphate group leaves an AMP unit with one metal ion. Hence, the question arises whether the remainder—that is, M_3_(ATP)(AMP)—is a reactive complex. Indeed, with M^2+^ = Cu^2+^, this is the case, as is shown in Figure 11.

In a Cu^2+^/ATP 2:1 system with [Cu]_tot_ = 2 × 10^−3^ M and [ATP]_tot_ = 10^−3^ M, the addition of AMP enhances the dephosphorylation rate. At an AMP concentration of about 7 × 10^−3^ M, the rate enhancement amounts nearly to a factor of four, and this demonstrates that AMP can take over the role of a structuring ATP. What are the reasons that the mixed stacks are formed? They are twofold: (i) The concentration of the mixed stacks is favored by statistics simply because the AMP concentration is larger than that of ATP, and (ii) the charge repulsion in a [(AMP)(ATP)]^6−^ stack is smaller than in an [(ATP)_2_]^8−^ one.

**Figure 12:**
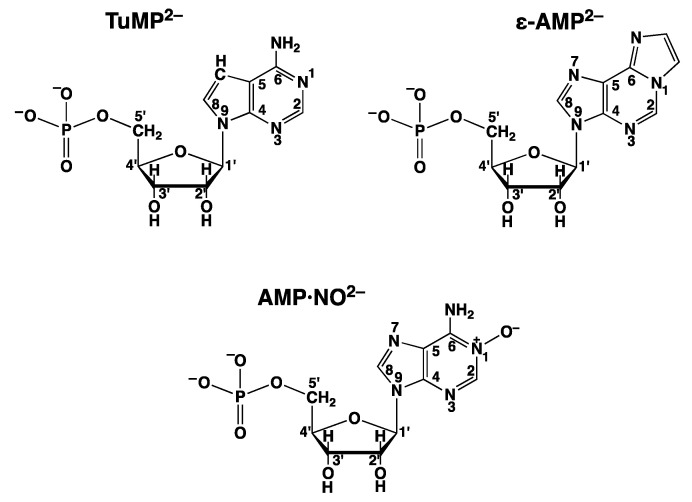
Chemical structures of AMP^2−^ analogues: tubercidin 5′-monophosphate (TuMP^2−^ = 7-deaza-AMP^2−^), 1,*N*^6^-ethenoadenosine 5′-monophosphate (ε-AMP^2−^), and adenosine 5′-monophosphate N(1)-oxide (AMP·NO^2−^).

That the M^2+^/N7 interaction in the stacks is crucial follows from experiments in which tubercidin 5′-monophosphate (TuMP^2−^ = 7-deaza-AMP^2−^; see Figure 12), which lacks N7 as this is replaced by CH, is added to the Cu^2+^/ATP 2:1 system, and this reduces the dephosphorylation rate considerably [14,80]. This is despite the structural identity and the equality of self-stacking of TuMP^2−^ and AMP^2−^ [6].

The structural sensitivity of the reactive intermediate (Figure 10) is further demonstrated by the addition of 1,N^6^-ethenoadenosine 5′-monophosphate (ε-AMP; see Figure 12) to the Cu^2+^/ATP 2:1 system, which reduces the dephopsphorylation rate drastically: At [ε-AMP]_tot_ = 4·10^−3^ M, the rate is practically zero (Figure 11). The stacking properties of ε-AMP^2−^ are very similar to those of AMP [95,96], but the coordination of Cu^2+^ to the 1,10-phenenthroline-like (C6)N,N7 site [95,96] leads to a different orientation of the metal ion in space and thus inhibits the dephosphorylation.

Similarly, adenosine 5′-monophosphate *N*(1)-oxide (AMP·NO; Figure 12) loses under the experimental conditions (which are given in the legend of Figure 11) a proton, and Cu^2+^ binds thus to the ionized *o*-amino N-oxide group [97,98,99]. This means that the reactive stack involving N7 (Figure 10) cannot be formed; the Cu^2+^ ion is wrongly orientated in space, and AMP·NO is thus a strong inhibitor of the dephosphorylation reaction [14,80].

In fact, all ligands that have a larger affinity for M^2+^ than the metal ion has for N7 will destroy the reactive intermediates seen in Figure 10. Hence, if one adds to the Cu^2+^/ATP 2:1 system at pH 6.7 (see legend of Figure 11) ligands such as phosphate or ATP, the reactivity will decrease [14]. Ligands such as *L*-tryptophan (trp) or 2,2′-bipyridine (bpy) will form ternary complexes such as M(trp)(ATP)^3−^ [100,101,102,103] or M(bpy)(ATP)^2−^ [104,105,106,107,108] and thus inhibit the dephosphorylation as well; indeed, bpy can even protect ATP against hydrolysis [109]. Ligands such as dien (diethylenetriamine = 1,4,7-triazaheptane) or dpa [di(2-picolyl)amine] will even remove in part the metal ions such as Cu^2+^ from the reactive complexes and will thus inhibit dephosphorylation strongly (see Figure 9 in [14] or Figure 22 in [11]).

In the preceding paragraphs and in the context of discussing the impact on the dephosphorylation rate of the AMP derivatives seen in Figure 12, it became evident that the slightest alteration in the structure of the adenine residue reduces the dephosphorylation rate. Evidently, these altered AMP derivatives are unable to take over the structuring role in the dimers (left site of the dimers in Figure 10). It was therefore interesting to see whether the acyclic AMP analogue, i.e., PMEA (Figure 5), had any effect on the reactivity [94].

PMEA owns an unaltered adenine residue; hence, one condition for activity is fulfilled and the question is: Can the phosphonate group at the aliphatic ether chain form with a metal ion a bridge and take over the structuring role? The answer is YES, as Figure 11 shows. Addition of about eight equivalents of PMEA to the Cu^2+^/ATP 2:1 system with [Cu^2+^]_tot_ = 2·10^−3^ M and [ATP]_tot_ = 10^−3^ M increases the dephosphorylation rate by a factor of nearly seven. Hence, Cu_3_(ATP)(PMEA) or better Cu_3_(ATP)(PMEA)(OH)^−^ (see Section 6.1) is even a more reactive species than Cu_3_(ATP)(AMP)(OH)^−^, which has an enhancement factor of “only” about four. The reason for this observation is clearly the larger flexibility of the aliphatic ether chain, compared with the rigid ribose residue of AMP.

### 6.4. Matrix-Assisted Self-Association and Its Significance for Cell Organelles with High ATP Concentrations

This section (with small alterations) is reproduced with permission from our publication in *Pure and Applied Chemistry* [21]; copyright 2004, International Union of Pure and Applied Chemistry (IUPAC).

The results summarized in Section 6.2 show that the extent of aggregation (as described by the isodesmic model of non-cooperative self-association (Equations (18) and (19)) “is much affected by external conditions such as concentration, pH or the presence of metal ions ([11,23,80,91,92,93]), the neutralization of the negative charges at the phosphate groups being an important factor for stack formation. This has prompted a study of the effect of poly-α,*l*-lysine, p(Lys)_n_, on the self-stacking properties of ATP in D_2_O [110,111]. At pD 8.4 all the ε-amino groups of the side chains of p(Lys)_n_ are protonated and carry a positive charge; i.e., ATP^4−^ with its negative phosphate groups is expected to “line up” along the ${p(H\cdot \mathrm{Lys})}_{n}^{n+}$ matrix. Indeed, under the mentioned conditions with [p-Lys]_side chains_ about 0.4 M and [ATP^4−^] ≤ 0.25 M, *K* = 11.5 ± 2.1 M^−1^ based on the isodesmic model [Equations (18) and (19)] [111]. This value should be compared with *K* = 1.3 ± 0.2 M^−1^ (Table 6) measured for ATP^4−^ in the absence of any promoter. That indeed the positively charged side chains of ${p(H\cdot \mathrm{Lys})}_{n}^{n+}$ are responsible for the increased stacking tendency is proven by the fact that at pD 12, where the side chains of p(Lys)_n_ are largely deprotonated, the stack-promoting effect of p(Lys)_n_ has largely disappeared [111].”

“The indicated self-association of ATP is important for certain cell organelles; for example, for the 5-hydroxytryptamine organelles of mammalian blood platelets it was estimated that among other solutes [ATP] is 0.5 M, [ADP] 0.1 M, [Mg^2+^] 0.44 M, and [Ca^2+^] 0.11 M [112,113]. Similar ATP concentrations appear to hold, e.g., for cholinergic, neurosecretory or synaptic vesicles and granules (see Refs and Table 4 in [111]). Clearly, such vesicles should be osmotically unstable [114,115,116], yet based on the summarized results one may conclude that the high nucleotide concentrations in such vesicles can be handled by nature via self-association and aggregate formation [114,115,116]. Indeed, e.g., the chromaffin granules of bovine adrenal medulla contain next to nucleotides (0.2 M; mainly ATP) and metal ions (0.03 M; mainly Mg^2+^ and Ca^2+^) [117,118] also so-called chromogranines, i.e., low-molecular-weight proteins which might be used as a matrix for aggregate formation”. (For further “Conclusions Regarding Other Matrices” see [111]).

**Figure 13:**
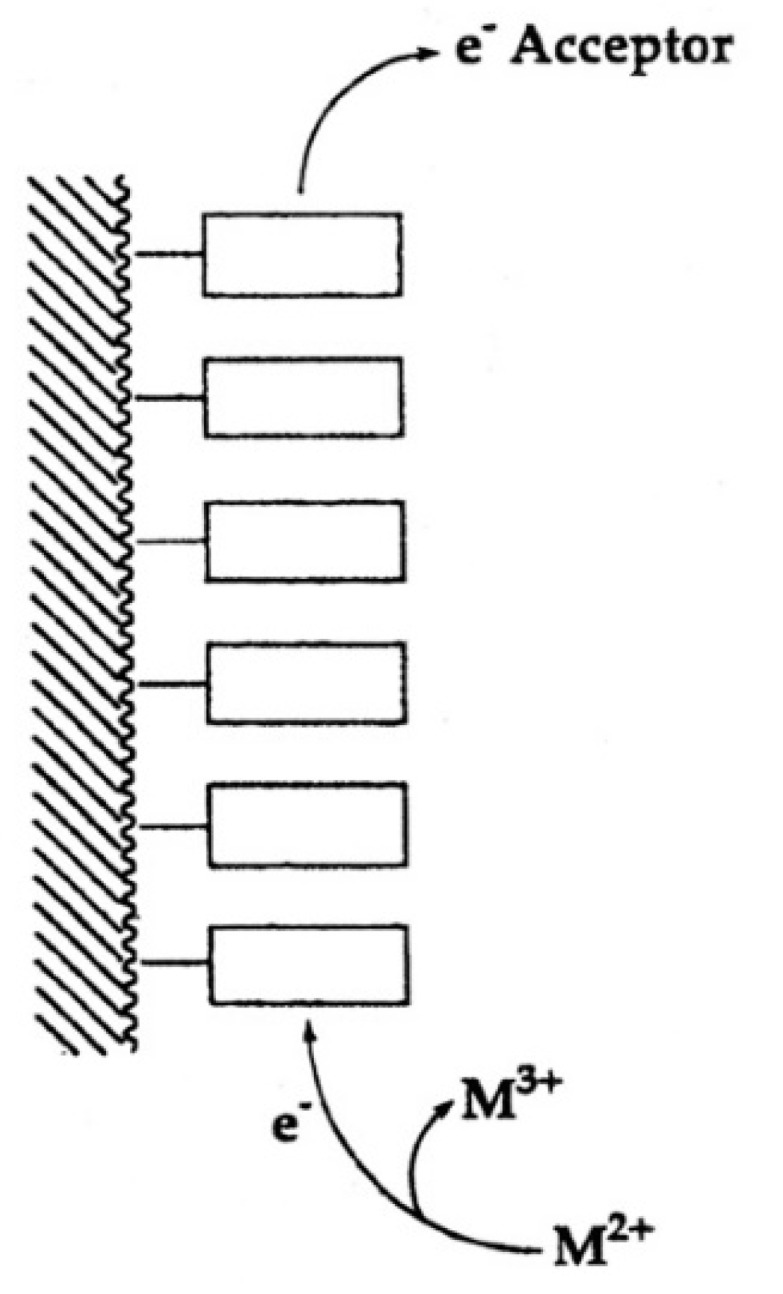
Simplified picture of a matrix-assisted ATP stack indicating an electron and thus an information transfer through this stack from one end to the other. This figure is reproduced with permission from our publication in *Pure and Applied Chemistry* [21]; copyright 2004, International Union of Pure and Applied Chemistry (IUPAC).

“In fact, one is tempted to speculate further: Considering that extracellular ATP is universally employed in cell-cell communication, particularly in synaptic transmission [119] (references therein) and if one recalls that electrons may migrate over long distances in DNA [120,121], one may propose that one way to achieve information transfer (and there are others) occurs in the following way. Assuming that, say six ATPs are lined up to form a stack covering a distance of approximately 20 Å one could imagine that at one end of the stack a metal ion (Fe^2+^, Mn^2+^, Cu^+^) is oxidized to a higher charged state (Fe^3+^, Mn^3+^, Cu^2+^) by a redox reaction and that this oxidized ion triggers hydrolysis of the triphosphate residue and that the electron travels through the stack to an acceptor at its other end [111]. This is depicted in a simplified fashion in Figure 13. That oxidation of a divalent metal ion (Mn^2+^) to a trivalent one (Mn^3+^) dramatically enhances the dephosphorylation rate of nucleoside 5′-triphosphates is known [11,73].”

## 7. Solution Structures of Mixed-Ligand Complexes Containing ATP^4−^ and Related Ligands

### 7.1. Ligands Containing a Ribose Residue with a Phosphate Group

Nearly 50 years ago, it was discovered by UV-spectrophotometry that a charge-transfer band occurs in mixed ligand Cu^2+^ complexes formed by AMP^2−^ or ATP^4−^ and a heteroaromatic amine (arm)—i.e., 2,2′-bipyridine (bpy) or 1,10-phenanthroline (phen) [104,122]. This charge-transfer band was attributed to the formation of an intramolecular stack, as indicated in Equilibrium (22):



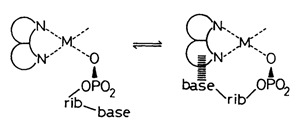

(22)


**Figure 14:**
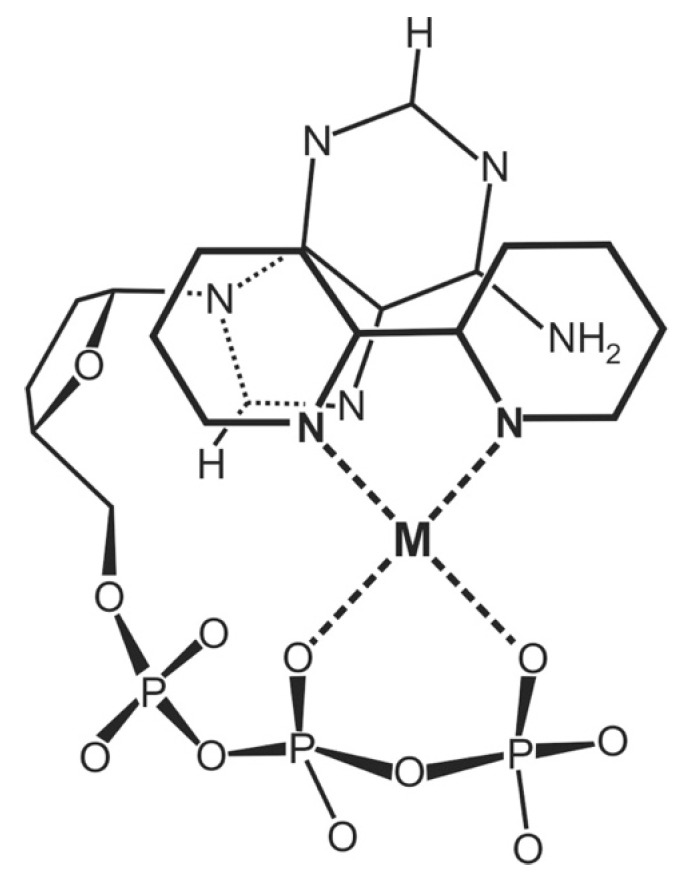
Probable schematic structure in solution of an intramolecular stack in the Cu(bpy)(ATP)^2−^ complex [105]; adapted from similar structures for Cu(bpy)(AMP), as shown in Refs [124,125,126].

From the fact that (i) the stability constant, e.g., of Cu(bpy)(ATP)^2−^, as determined from potentiomtric pH titrations on the one hand (log $K_{\mathrm{Cu}(\mathrm{bpy})(\mathrm{ATP})}^{\mathrm{Cu}(\mathrm{bpy})}$ = 6.91 ± 0.15 [123]) and based on the charge-transfer band on the other (log $K_{\mathrm{Cu}(\mathrm{bpy})(\mathrm{ATP})}^{\mathrm{Cu}(\mathrm{bpy})}$ = 6.96 ± 0.25 [104]) were practically identical and that (ii) also the extinction coefficient of the binary ATP^4−^/bpy adduct was similar to the one of the corresponding ternary complex indicated that the formation degree of the intramolecular stack in Cu(bpy)(ATP)^2−^ is high; a tentative structure is shown in Figure 14 [104,124,125,126].

Of course, the stability of the binary ATP^4−^/bpy stacking adduct is low (log *K*_CT_ = 0.91 ± 0.22) [104]; addition of a metal ion bridges the two ligands, and the stability increases, as is known from many examples [101,106,127,128,129,130]. Indeed, for example, the formation degree of the intramolecular stack in the ternary Cu(bpy)(AMP) and Cu(phen)(AMP) complexes is high—it amounts to 81 (±4) and 90 (±2)%, respectively [125]. Finally, solids of such complexes were isolated, and by X-ray crystal structure studies, also intramolecular stacking interactions were proven [107,108,131].

**Figure 15:**
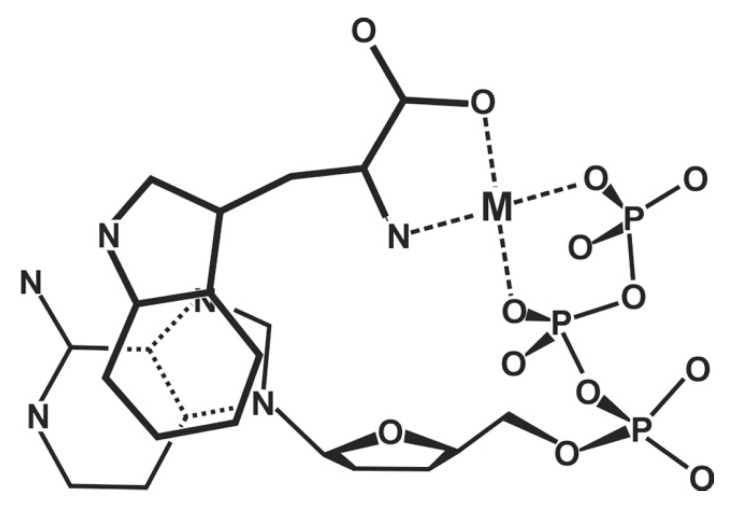
Tentative and schematic structure of the stacked isomer in solution of an M(ATP)(Trp)^3−^ complex. Adapted from Ref [101].

“The first mixed ligand complex containing ATP and an amino acid was one with tryptophanate, i.e., Zn(ATP)(Trp)^3−^. By ^1^H NMR shift experiments it was shown that an indole-adenine interaction takes place [132] which may be promoted by Zn^2+^ (see also [101]). Later, the position of the intramolecular Equilibrium (23)

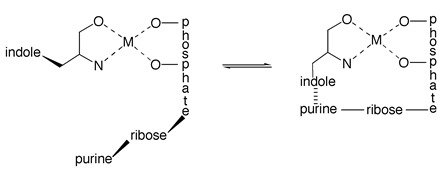
(23)
was determined with Zn^2+^ as metal ion and it was concluded [103] that the stacked species occurs with a formation degree of approximately 75%. Other metal ions were studied as well [100,103,133,134] and the occurrence of intramolecular stacks in M(ATP)(Trp)^3−^ complexes” (for a tentative structure [101] see Figure 15) “was confirmed by several groups [135,136,137,138]. Related complexes containing phenyl and imidazole residues have also been investigated [124,139,140].”

“Moreover, it was shown that the isopropyl residue of leucinate is also able to undergo a hydrophobic interaction in M(ATP)(Leu)^3−^ complexes; e.g., the formation degree of the “closed” species of the corresponding Mn^2+^ complex amounts to about 40% [103]. The occurrence of such species with a hydrophobic interaction was again proven by ^1^H NMR shift studies [103] and by comparisons of stability constants, which were mostly determined via potentiometric pH titrations [103]” (see also [126]).

“Quantification of the formation of *intra*molecular stacks in mixed ligand complexes containing a heteroaromatic amine (Arm) allowed the conclusion [126] that recognition between the adenine residue and amino acid side chains in mixed ligand complexes of the type M(ATP)(aa)^3−^ (where aa = amino acetate derivative) decreases in the order (partly tentative): indole residue (tryptophan) > phenyl residue (phenylalanine) ≥ isopropyl residue (leucine) ≥ imidazole residue (histidine) > methyl residue (alanine).”

The preceding three paragraphs (with small alterations are reproduced with permission from our publication in *Pure and Applied Chemistry* [21]; copyright 2004, International Union of Pure and Applied Chemistry (IUPAC).

### 7.2. Acyclic Nucleoside Phosphonates: Cu(arm)(PMEA) as an Example with Intramolecular Stack Formation

**Figure 16:**
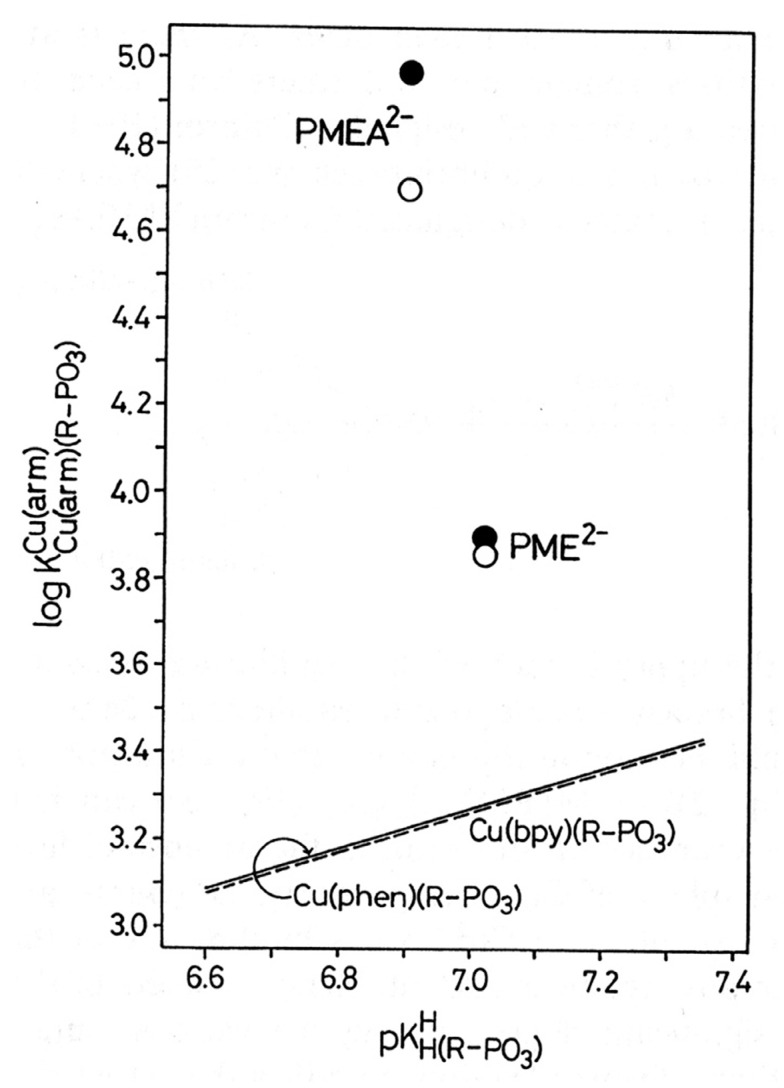
Evidence for an enhanced stability of the Cu(arm)(PME) and Cu(arm)(PMEA) complexes based on the relationship between log $K_{{\mathrm{Cu}(\mathrm{arm})(R-\mathrm{PO}}_{3})}^{\mathrm{Cu}(\mathrm{arm})}$ and ${pK}_{{H(R-\mathrm{PO}}_{3})}^{H}$ for the ternary. Cu(bpy)(PME) and Cu(bpy)(PMEA) (◯), as well as for the Cu(phen)(PME) and Cu(phen)(PMEA) (●) complexes in aqueous solution at *I* = 0.1 M (NaNO_3_) and 25 °C. The plotted data are from Table 6 in Section 8 of [141] (= Table 7 here). The two reference lines represent the log *K* versus p*K*_a_ relationship for Cu(arm)(R-PO_3_) complexes; it should be emphasized that R-P$O_{3}^{2–}$ symbolizes here phosphonates (or phosphate monoesters) with an R group unable to undergo any kind of hydrophobic, stacking, or other type of interaction; the broken line holds for arm = bpy and the solid line for arm = phen. Both straight lines are calculated with the parameters listed in Table 5 of Ref [141], and they represent the situation for ternary complexes without an intramolecular ligand–ligand interaction. Redrawn in a slightly altered way from Ref [141] with permission of the Royal Society of Chemistry and then reproduced with permission from our publication in *Coordination Chemistry Reviews* [45]; copyright 1995, Elsevier Science S.A., Lausanne, Switzerland.

In Section 6.2, we have seen that aromatic moieties can form stacks and that this holds quite generally [125]—e.g., also for 1,10-phenanthroline [141]. Therefore, it is important to point out that all results described below were obtained under conditions where self-association was negligible, and the results refer thus to monomeric species [45,141]. That in Cu(arm)(PMEA) complexes the adenine residue affects the stability of the complexes in a positive manner is immediately evident from Figure 16 [141].

**Figure 17:**
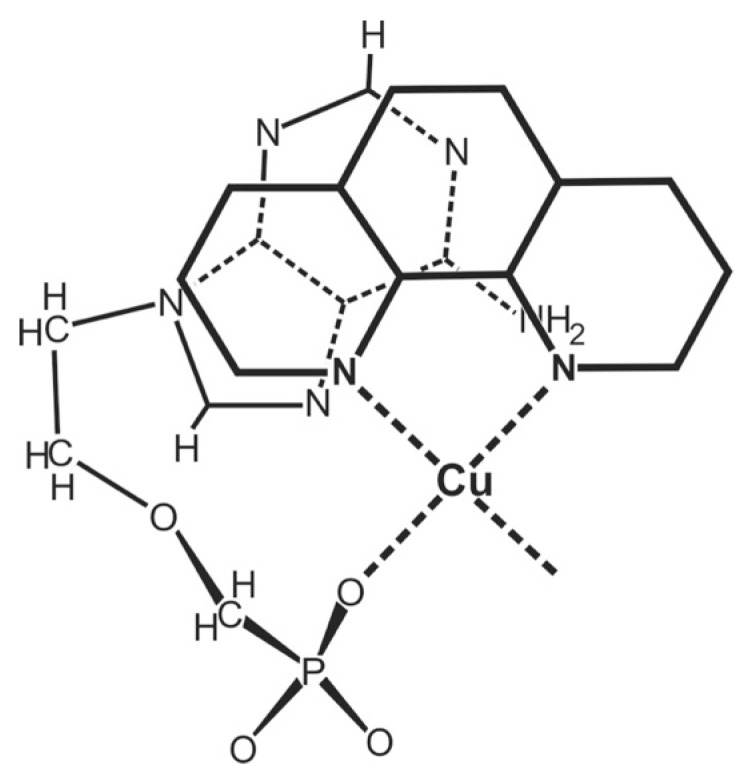
Tentative and simplified structure of a species with an intramolecular stack for Cu(phen)(PMEA) in solution [45,141]. Adapted from Ref [45].

With arm coordinated to Cu^2+^, there are only two equatorial coordination sites left for binding of a second ligand. Because in PMEA^2−^ the phosphonate group is the primary binding site (Section 3), Equilibrium (5), which involves the ether O atom, is of relevance also for the ternary complexes, but a further direct equatorial coordination of the adenine residue is not possible. Hence, the only remaining way how the adenine residue can affect complex stability is via the formation of an intramolecular stack (Figure 17) [45,141].

Careful considerations of the steric situation [45,141] reveal that the formation of an intramolecular stack is only possible if the ether oxygen (Equation (5)) is released from the coordination sphere; only this provides enough flexibility. Aside from the open isomer, Cu(arm)(PMEA)_op_, there are two more isomers, one that involves the ether O atom, Cu(arm)(PMEA)_cl/O_ and one with the intramolecular stack, Cu(arm)(PMEA)_st_; this then gives rise [45,141] to Equilibrium Scheme (24):



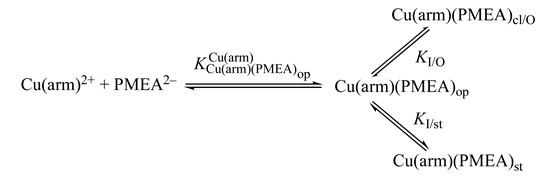

(24)


To determine the formation degrees of the various species, one has to recall that the total stability increase is experimentally accessible and that the stability of the open isomers can be calculated via the reference lines seen in Figure 16; this leads to the following equations [45]:

(25) $$K_{{\mathrm{Cu}(\mathrm{arm})(\mathrm{PMEA})}_{\mathrm{op}}}^{\mathrm{Cu}(\mathrm{arm})}=[\mathrm{Cu}(\mathrm{arm})(\mathrm{PMEA}{)}_{\mathrm{op}}]/([\mathrm{Cu}{\left(\mathrm{arm}\right)}^{2+}][{\mathrm{PMEA}}^{2-}])$$

*K*_I/O_ = [Cu(arm)(PMEA)_cl/O_]/[Cu(arm)(PMEA)_op_] (26)

*K*_I/st_ = [Cu(arm)(PMEA)_st_]/[Cu(arm)(PMEA)_op_] (27)

For the experimentally accessible equilibrium constant it holds,

(28) $$\begin{array}{c} K_{\mathrm{Cu}(\mathrm{arm})(\mathrm{PMEA})}^{\mathrm{Cu}(\mathrm{arm})}=\frac{\left[\mathrm{Cu}(\mathrm{arm})(\mathrm{PMEA})\right]}{{\left[\mathrm{Cu}(\mathrm{arm}\right)}^{2+}{][\mathrm{PMEA}}^{2–}]}\\ \;\;=\frac{{\left([\mathrm{Cu}(\mathrm{arm})(\mathrm{PMEA}\right)}_{\mathrm{op}}{]+[\mathrm{Cu}(\mathrm{arm})(\mathrm{PMEA})}_{\mathrm{cl}/O}{]+[\mathrm{Cu}(\mathrm{arm})(\mathrm{PMEA})}_{\mathrm{st}}])}{{\left[\mathrm{Cu}(\mathrm{arm}\right)}^{2+}{][\mathrm{PMEA}}^{2–}]}\\ \;\;=K_{{\mathrm{Cu}(\mathrm{arm})(\mathrm{PMEA})}_{\mathrm{op}}}^{\mathrm{Cu}(\mathrm{arm})}+K_{I/O}\cdot K_{{\mathrm{Cu}(\mathrm{arm})(\mathrm{PMEA})}_{\mathrm{op}}}^{\mathrm{Cu}(\mathrm{arm})}+K_{I/\mathrm{st}}\cdot K_{{\mathrm{Cu}(\mathrm{arm})(\mathrm{PMEA})}_{\mathrm{op}}}^{\mathrm{Cu}(\mathrm{arm})}\\ \;\;=K_{{\mathrm{Cu}(\mathrm{arm})(\mathrm{PMEA})}_{\mathrm{op}}}^{\mathrm{Cu}(\mathrm{arm})}(1+K_{I/O}+K_{I/\mathrm{st}}) \end{array}$$

and for the intramolecular interactions one obtains

(29) $$\begin{array}{c} K_{I}=K_{I/\mathrm{tot}}=\frac{K_{\mathrm{Cu}(\mathrm{arm})(\mathrm{PMEA})}^{\mathrm{Cu}(\mathrm{arm})}}{K_{{\mathrm{Cu}(\mathrm{arm})(\mathrm{PMEA})}_{\mathrm{op}}}^{\mathrm{Cu}(\mathrm{arm})}}={10\log\;}^{\Delta }-1\\ \;=\frac{{\left[\mathrm{Cu}(\mathrm{arm})(\mathrm{PMEA}\right)}_{\mathrm{int}/\mathrm{tot}}]}{{\left[\mathrm{Cu}(\mathrm{arm})(\mathrm{PMEA}\right)}_{\mathrm{op}}]}\\ \;=\frac{{\left([\mathrm{Cu}(\mathrm{arm})(\mathrm{PMEA}\right)}_{\mathrm{cl}/O}{]+[\mathrm{Cu}(\mathrm{arm})(\mathrm{PMEA})}_{\mathrm{st}}])}{{\left[\mathrm{Cu}(\mathrm{arm})(\mathrm{PMEA}\right)}_{\mathrm{op}}]}\\ \;=K_{I/O}+K_{I/\mathrm{st}} \end{array}$$

Clearly, Cu(arm)(PMEA)_int/tot_ in Equation (29) represents the sum of both species with an intramolecular interaction.

Since the overall stability constant as defined by Equation (28) is experimentally accessible (Table 7, Column 2), and because the stability constant of the open isomer (Equation (25)), in which only the phosphonate group is coordinated (Table 7, Column 3), can be calculated with the information given in Figure 16, the stability enhancement log *Δ*, as defined by Equation (12)), is obtained (Table 7, Column 4). These log *Δ* values can be used with Equation (29) to calculate *K*_I/tot_ (Table 7, Column 5), and from these data follow (Equation (14)) the formation degrees in percentage for Cu(arm)(PMEA)_int/tot_ (Table 7, Column 6).

**Table 7:** Quantification of the stability increase via log *Δ* (analogous to Equation (12)) for the Cu(arm)(PME) and Cu(arm)(PMEA) complexes and extent of the intramolecular chelate formation (Equation (14)) in the Cu(arm)(PME) complexes at 25 °C and *I* = 0.1 M (NaNO_3_); the results for the corresponding binary Cu(PME) complex are given for comparison.

M^2+^	log $\;K_{M(\mathrm{PME})}^{M}$ (Equations (8), and (28)) ^*a*^	log $\;K_{{M(\mathrm{PME})}_{\mathrm{op}}}^{M}$ (Analogous to Equation (25)) ^*b*^	log *Δ*_PME_ (Equation (12)) ^c^	*K*_I_ (Equations (6), (9) and (29)) *^c^*	% M(PME)_cl_ (Equation (14)) *^c,d^*
Cu^2+^	3.73 ± 0.03	3.25 ± 0.06	0.48 ± 0.07	2.02 ± 0.47	67 ± 5
Cu(bpy)^2+^	3.86 ± 0.03	3.27 ± 0.07	0.59 ± 0.08	2.89 ± 0.68	74 ± 5
Cu(phen)^2+^	3.90 ± 0.04	3.28 ± 0.06	0.62 ± 0.07	3.17± 0.69	76 ± 4
**M^2+^**	**log ** $\;K_{M(\mathrm{PMEA})}^{M}$ **[Equation (28)] *^a^***	**log ** $\;K_{{M(\mathrm{PMEA})}_{\mathrm{op}}}^{M}$ **[Equation (25)] *^b^***	**log *Δ*_PMEA_** **[Equation (12)] *^c^***	***K*_I_ = *K*_I/tot_** **[Equation (29)] *^c^***	**% Cu(arm)(PMEA)_int/tot_** **[Equation (29); analog. to Equation (14)]**
Cu(bpy)^2+^	4.70 ± 0.02	3.22 ± 0.07	1.48 ± 0.07	29.20 ± 4.87	96.69 ± 0.53
Cu(phen)^2+^	4.97 ± 0.03	3.23± 0.06	1.74 ± 0.07	53.95 ± 8.86	98.18 ± 0.29

*^a^* The constants were measured in aqueous solution via potentiometric pH titrations [44]. The constants are from [141]. For the error limits see Footnote ‘*a*’ of Table 2. *^b^* These constants were calculated with the p*K*_a_ values 7.02 and 6.90 for H(PME)^−^ and H(PMEA)^−^, respectively (see footnotes in Table 2) and the straight-line equations given in Table 5 of Ref [141]; the corresponding error limits are 3 times the SD values (see Table 5 in Ref [141]). *^c^* The error limits for log *Δ*, *K*_I_, and % M(PME)_cl_ were calculated according to the error propagation after Gauss; the same applies for the lower part of the table. *^d^* See Equilibrium (5). This table is reproduced (slight alterations were made) with permission from Table 8 in our publication in *Coordination Chemistry Reviews* [45]; copyright 1995, Elsevier Science S.A., Lausanne, Switzerland.

Based on the justified assumption that the stability of Cu(arm)(PME)_cl_ equals that of Cu(arm)(PMEA)_cl/O_, one obtains *K*_I/O_ for the latter species (Table 8, Column 5), and thus from Equation (29) *K*_I/st_ follows (see Table 8, Column 6) because *K*_I/tot_ is known. Therefore, the formation degrees of all three isomers that occur in Equilibrium Scheme (24) are known (Table 8, Columns 4, 7, and 8).

First, it should be noted that all three isomers according to Equilibrium Scheme (24) occur, but that the open isomer is a minority species for both Cu(arm)(PMEA)_op_ complexes: The Cu(bpy)(arm)_cl/O_ and the Cu(phen)(arm)_cl/O_ species form to about 10% and 6%, respectively. The dominating species are clearly the one with the intramolecular stack, Cu(arm)(PMEA)_st_, which reach a formation degree of about 90% (Table 8, Column 8). This high formation degree of the stacked species suppresses the formation of the 5-membered chelate of Equilibrium (5); this contrasts with Cu(arm)(PME)_cl_, which reaches a formation degree of about 75% (Table 7, upper part, Column 6).

With regard to biological systems, one may note that it is expected that intramolecular stacks in ternary complexes do not play a significant role. However, what follows from the high formation degree of the Cu(arm)(PMEA)_st_ species is that the adenine residue provides a good anchoring point. Such a point could be created by stack formation with another nucleobase, e.g., of a ribozyme, or by stack formation with an aromatic amino acid side chain of a protein. Such stacks can then give rise to the formation of reactive complexes, as seen in Figure 2; that is, the coordination of two metal ions to a triphosphate can occur either via M(α,β)-M(γ) or via M(α)-M(β,γ), leading to different products.

## 8. The Effect of a Change in Solvent Polarity on the Stability and Structure of Binary and Ternary Complexes

### 8.1. The Dianion of (Phosphonomethoxy)ethane (PME^2−^) as an Example of a Polar O-Ligand. The Properties of Cu(PME) Are Largely as Expected

A change in solvent polarity is expected to affect especially polar or ionic interactions. For PME^2−^, this means, as follows from Equilibrium (5), metal ion binding to the phosphonate group and the ether oxygen site is likely to be altered. Indeed, these interactions are expected to be affected by the so-called “effective” or “equivalent solution” dielectric constant. This constant is reduced in proteins [142,143,144] and in active site cavities of enzymes [145], if compared with bulk water; hence, the activity of water is reduced [146]. For example, the effective dielectric constant in the active sites of bovine carbonic anhydrase and carboxypeptidase A is 35 and <70, respectively [145], compared with about 78.5 for bulk water at 25 °C [147,148]. By applying 1,4-dioxane-water mixtures, one may expect to simulate to some extent the situation in an active site cavity; the dielectric constants are about 53 and 35 in water containing 30% or 50% (*v*/*v*) 1,4-dioxane, respectively [147,148].

**Figure 18:**
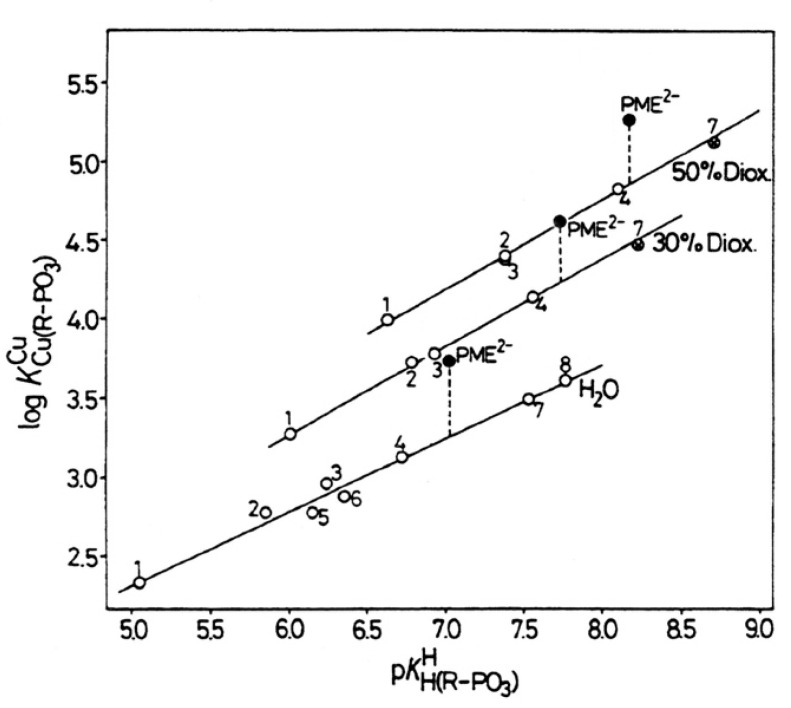
Evidence for an enhanced stability of the Cu(PME) (●) complex in 1,4-dioxane-water mixtures as solvents, based on the relationship between log $K_{{\mathrm{Cu}(R-\mathrm{PO}}_{3})}^{\mathrm{Cu}}$ and ${pK}_{{H(R-\mathrm{PO}}_{3})}^{H}$ for the Cu^2+^ 1:1 complexes of 4-nitrophenyl phosphate (1), phenyl phosphate (2), D-ribose 5-monophosphate (3), *n*-butyl phosphate (4),uridine 5′-monophosphate (5), thymidine [= 1-(2′-deoxy-β-D-ribofuranosyl)thymine] 5′-monophosphate (6), methanephosphonate (7), and ethanephosphonate (8) in water and in water containing 30% or 50% (*v*/*v*) 1,4-dioxane. The least-squares lines are drawn in each case through the data sets shown [44,150]; the equations for these reference lines are given in Ref [149]. The data points due to the methanephosphonate system in the mixed solvents (⊗,7) (see Ref [149]) are shown to prove that simple phosphonates fit within the experimental error limits on the reference lines established with phosphate monoester systems (see also Ref [149). The points due to the Cu^2+^ 1:1 complexes formed with PME^2−^ (●) in the three mentioned solvents demonstrate the enhanced complex stabilities. The vertical broken lines emphasize the stability differences to the reference lines; these differences equal log *Δ*_Cu(R-PO__3)_, as defined in Equation (12). All the plotted equilibrium constants refer to 25 °C and *I* = 0.1 M (NaNO_3_). This figure is adapted with permission from our publication in *Inorganic Chemistry* [149]; copyright 1993, American Chemical Society (see also [45]).

To address the above situation, log $K_{{\mathrm{Cu}(R-\mathrm{PO}}_{3})}^{\mathrm{Cu}}$ versus ${pK}_{{H(R-\mathrm{PO}}_{3})}^{H}$ plots for water and water containing 30% or 50% (*v*/*v*) 1,4-dioxane were established [149,150]; the corresponding straight-line references are shown in Figure 18 [44,45,149,150].

It is evident that with increasing 1,4-dioxane concentration, the stability of the Cu^2+^-phosphonate complexes increases; at ${pK}_{{H(R-\mathrm{PO}}_{3})}^{H}$ = 7.0, this increase amounts to about 0.57 and 0.93 log units by the solvent change from water to water containing 30% or 50% (*v*/*v*) 1,4-dioxane; these are stability increases by factors of nearly 4 and 10, respectively. The properties of these solvents are listed in Columns 2 to 4 of Table 9. To conclude, the Cu^2+^-phosphonate binding is clearly facilitated by a decreasing solvent polarity, as one would have expected.

The data points for the Cu(PME) complex (●) are in all three solvents significantly above the reference lines. It should be recalled that the vertical differences to the straight-reference lines correspond to the stability enhancements log *Δ*_Cu(PME)_ (Equation (12)). These values are listed in Column 5 of Table 9; application of these data to Equation (10) provides *K*_I_ (Table 9, Column 6) and thus with Equation (14) also the formation degrees of the closed species are obtained (Table 9, Column 7). It is interesting to note that the arithmetic means of log *Δ*_Cu(PME)_ (0.43 ± 0.07) and % Cu(PME)_cl_ (62 ± 3) overlap within the error limits with the individual results and that this seems to indicate that the size of the stability enhancements log *Δ*_Cu(PME)_ and the formation degrees % Cu(PME)_cl_ appear largely to be independent of the solvent. This may seem somewhat surprising, especially in the light of the overall stability increase in the Cu(PME) complexes (Figure 18) with the decreasing solvent polarity. On the other hand, this result implies that the solvent effect is about the same for both binding sites, the phosphonate group, and the ether oxygen of the aliphatic chain.

### 8.2. Nitrogen Donor Sites Are Especially Sensitive to Polarity Changes of the Solvent

With the observations of the oxygen-binding sites described in the preceding Section 8.1 in mind, it seems appropriate to consider now ligands with N-sites, as such sites are of relevance for biological systems as well. Some of such results are compiled in Table 10 [127].

For the planar 1,10-phenanthroline (phen) with its three fused aromatic 6-membered rings, one expects self-association. This is in aqueous solution very pronounced indeed (Table 10; Entry (1.a)), but it drops with 2,2′-bipyridine (bpy) to about one fourth due to the removal of the bridging aromatic ring (Table 10; Entry (1.b)). For both N-ligands, the self-stacking tendency decreases very significantly by the addition of 1,4-dioxane to the aqueous solution. This indicates that the ethylene units of dioxane solvate the aromatic rings of phen and bpy and suppress in this way self-stacking.

The results just indicated for the self-stacking process are reflected in the stability constants $K_{\left(\mathrm{arm})(\mathrm{ATP}\right)}^{\left(\mathrm{ATP}\right)}$ observed for the binary stacking adducts (phen)(ATP)^4−^ and (bpy)(ATP)^4−^ (Table 10; Entries (2.a) and (2.b)). The adenine residue of ATP^4−^ is well suited to form stacks (see Section 7). That the adduct with phen in D_2_O is about twice as stable as the one with bpy fits into the picture, as does the dramatic decrease in the stability constants $K_{\left(\mathrm{arm})(\mathrm{ATP}\right)}^{\left(\mathrm{ATP}\right)}$ of the binary adducts by the addition of dioxane.

What happens if the two ligands ATP^4−^ and arm (= phen or bpy) of Entries (2.a) and (2.b) are bridged by a metal ion? This question is best answered by considering the *Δ* log *K*_Cu/arm/ATP_ values that are defined in Equation (30) and that quantify Equilibrium (31) and therefore also the stability of the ternary complexes:

(30) $$\Delta \;\log\;K_{\mathrm{Cu}/\mathrm{arm}/\mathrm{ATP}}=\log\;K_{\mathrm{Cu}(\mathrm{arm})(\mathrm{ATP})}^{\mathrm{Cu}(\mathrm{arm})}-\log\;K_{\mathrm{Cu}(\mathrm{ATP})}^{\mathrm{Cu}}\;\;\;\;\;\;\;\;=\log\;K_{\mathrm{Cu}(\mathrm{ATP})(\mathrm{arm})}^{\mathrm{Cu}(\mathrm{ATP})}-\log\;K_{\mathrm{Cu}(\mathrm{arm})}^{\mathrm{Cu}}$$

(31) $$\mathrm{Cu}{\left(\mathrm{arm}\right)}^{2+}+\mathrm{Cu}{\left(\mathrm{ATP}\right)}^{2-}\;⇌\mathrm{Cu}(\mathrm{arm}){\left(\mathrm{ATP}\right)}^{2-}+{\mathrm{Cu}}^{2+}$$

For *Δ* log *K*_Cu/arm/ATP_, one expects for statistical reasons a negative value: For the Jahn–Teller distorted Cu^2+^ and two bidentate ligands, *Δ* log *K*_Cu/statist_ ≅ –0.9 was estimated [127]. The results listed in Table 10 in Entries (3.a) and (3.b) for water are thus a surprise because they mean that Equilibrium (31) is shifted towards its right side. That the value for Cu(bpy)(ATP)^2−^ is smaller than the one for Cu(phen)(ATP)^2−^ is expected by having the results for Entries (2.a) and (2.b) in mind. Addition of dioxane affects the stability of the intramolecular stacks in Cu(arm)(ATP) in a negative manner, shifting Equilibrium (31) to the left, a result in accord with this given in Entries (2.a) and (2.b).

The two ligands of which Cu(arm)(ATP)^2−^ is composed need to be considered also in an indirect manner. For phen and bpy, this has already been carried out (Table 10; Entries (1.a) and (1.b)), and for ATP^4−^ (see also Table 6) it is made in the form of the Cu(ATP)^2−^ complex (Table 10; Entry (4.b)). This result is compared with that for Cu(UTP)^2−^, UTP^4−^ being known as having a very small tendency for self-association [127]. Addition of dioxane to Cu(UTP)^2−^ (Table 10; Entry (4.a)) increases the stability of the complex, and the three available data sets fit in a log $K_{\mathrm{Cu}(\mathrm{UTP})}^{\mathrm{Cu}}$ versus ${pK}_{H(\mathrm{UTP})}^{H}$ plot on a straight line [127]. This means that the stability of Cu(UTP)^2−^ increases, like the one of simple Cu^2+^-phosph(on)ate complexes [150,151], with decreasing solvent polarity and behaves thus in a normal manner.

This observation with Cu(UTP)^2−^ contrasts strongly with the one for Cu(ATP)^2−^, the stability of which is practically independent of the solvent and also larger in all three solvents (Table 10; Entry (4.b)) than is expected on the basis of the basicity of the phosphate residue. This basicity is the same in water for UTP^4−^ (${pK}_{H(\mathrm{UTP})}^{H}$ = 6.46 ± 0.01) and ATP^4−^ (${pK}_{H(\mathrm{ATP})}^{H}$ = 6.49 ± 0.01) [127]; this identity of the basicity also holds for the mixed solvents [127]. Hence, the increased stability of Cu(ATP)^2−^ in water must be due to the well-known [21,46,53,95] macrochelate formation with N7. This N7 interaction is inhibited by solvation of dioxane of the adenine residue hindering the coordination of Cu^2+^ to N7. Hence, the extent of the N7 interaction decreases with increasing amounts of dioxane, but in contrast, the Cu^2+^-phosphate binding strength increases. In other words, we have two opposing solvent effects that cancel each other and give rise to the observed (Table 10; Entry (4.b)) solvent-independent stability of the Cu(ATP)^2−^ complex.

### 8.3. Competing Solvent Effects on N- versus O-Sites in the Same Ligand and Its Bearing on Complex Stability

After the conclusion in the preceding Section 8.2 that N- and O-sites face opposing solvent effects, it is of interest to see how a ligand with such ambivalent properties affects complex stability upon a change in solvent polarity. An ideal ligand in this respect is the well-studied AMP^2−^ (Figure 3), which offers for coordination next to the primary binding site—hat is, the phosphate groups—the N7 site of the adenine residue that is involved in macrochelate formation (see Footnote ‘*c*’ in Table 11). The available data for the anion of the AMP analogue 9-[2-(phosphonomethoxy)ethyl]adenine (PMEA^2−^; Figure 3) are given for comparison. The values for the complexes of both ligands are listed in Table 11 [21,45,46,50,53,95,149,152,153].

The log stability constants of the Cu(AMP) complex (Table 11; Column 3) increase with increasing dioxane concentration, as expected for a ligand with a phosphate group (Section 3) as primary coordination site. The behavior of Cu(PMEA) is analogous: It is interesting to note that the stability enhancement by going from water to 50% (*v*/*v*) dioxane is identical for the two complexes—that is, it amounts for Cu(AMP) to 1.59 ± 0.04 and for Cu(PMEA) to 1.58 ± 0.08 log units. This confirms the close relationship between phosphate and phosphonate ligands. The corresponding differences in stability for the open isomers Cu(AD)_op_ are within the error limits identical with the mentioned ones (Table 11; Column 4). Most interesting, however, are the percentages of the macrochelates, Cu(AMP)_cl/N7_ (Table 11; Column 7): They pass, beginning with a formation degree of 46 ± 10% in water, through a minimum of 9 ± 8% in 30 % (*v*/*v*) dioxane, and then go up again to 48 ± 5% in 50% dioxane. This reflects exactly the discussed opposing solvent effects and thus the ambivalent nature of the AMP^2−^ ligand with its phosphate and N7 sites. The properties of Cu(PMEA) are analogous, though the stability differences are less pronounced (see the three bottom lines in Table 11).

**Figure 19:**
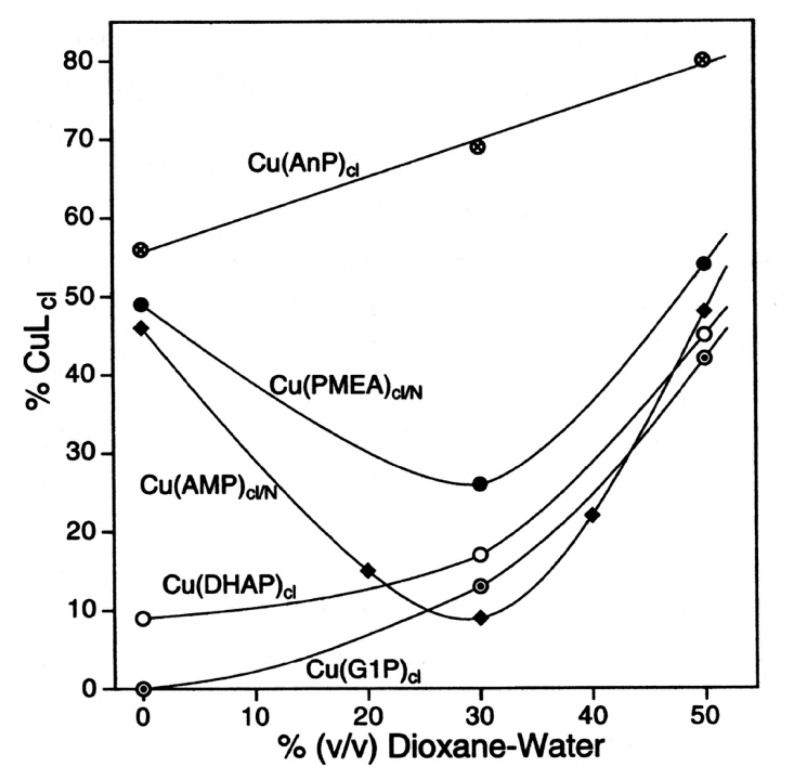
Formation degrees of the chelated isomers, CuL_cl_, in the Cu(AMP) and Cu(PMEA) systems (Table 11) as well as for comparison in the Cu(DHAP) [47], Cu(G1P) [47], and Cu(AnP) [41,47,154] complex systems (Figure 20) as a function of the percentage of 1,4-dioxane added to an aqueous reagent mixture. All the plotted equilibrium constants refer to 25 °C and *I* = 0.1 M (NaNO_3_). This figure is reproduced with permission from our publication in *Coordination Chemistry Reviews* [47]; copyright 2000, Elsevier, Science S.A., Lausanne, Switzerland).

As noted, weak interactions between metal ions and oxygen donor sites on the one hand and nitrogen donors on the other are very differently affected by a decreasing polarity of the solvent. This is nicely confirmed by the contents of Table 11 and illustrated in Figure 19, where the curves for % Cu(AMP)_cl_ and % Cu(PMEA)_cl_ pass through a minimum each, demonstrating again the ambivalent properties of these two ligands [41,47,154].

**Figure 20:**
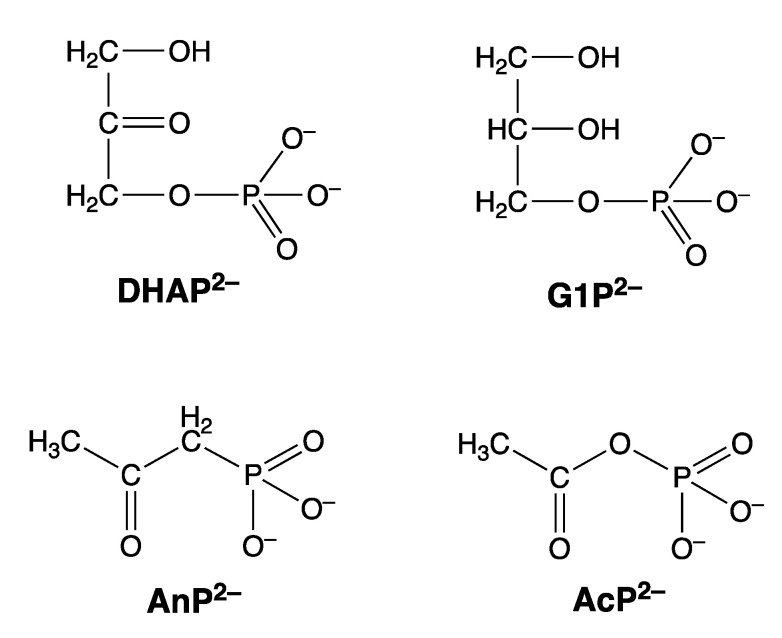
Chemical structures of dihydroxyacetone phosphate (DHAP^2−^) and glycerol 1-phosphate (G1P)^2−^. Acetonylphosphonate (AnP^2−^) is often employed as a model compound for acetyl phosphate (AcP^2−^), which is biologically very relevant but difficult to study because it is very hydrolysis-sensitive. It may be added that in the so-called [155] α-glycerophosphate shuttle, DHAP^2−^ and G1P^2−^ are interconverted into each other [155,156].

The ligand structures of the other Cu^2+^ complexes that appear in Figure 19 are pure O-donor ligands, as is seen in Figure 20 [155,156]. In all instances is the phosph(on)ate group the primary and thus the stability-determining binding site, but they all offer for weak interactions in their Cu^2+^ complexes either a carbonyl (DHAP^2−^, AnP^2−^, AcP^2−^) or a hydroxyl group (G1P^2−^).

In aqueous solution, the extent of this weak interaction varies strongly [47]: In Cu(AcP) the closed isomer amounts to 76 ± 4% and in Cu(AnP) to 56 ± 7% (also other M^2+^ reach high formation degrees), whereas in Cu(DHAP) and Cu(G1P) at best only traces (9 ± 13% for Cu(DHAP)) of the closed form exist. The reason for this different behavior is that in the case of Cu(AcP/AnP) 6-membered and in the case of Cu(DHAP/G1P) 7-membered chelates form. However, in the presence of dioxane, the formation degree of the chelates increases, and in 50% (*v*/*v*) 1,4-dioxane with Cu(DHAP) and Cu(G1P) 45 ± 4 and 42 ± 5%, respectively, of the complexes are present as 7-membered chelates [47]. For Cu(AnP) 80 ± 2% form 6-membered chelates [47]. In Figure 19, the corresponding plots are seen: In all three cases for which data exist, there is no minimum observed, but the curves increase with increasing dioxane concentration. This is as it should be for complexes of ligands that offer only O-donor sites.

To conclude, the discrimination that is achieved between N and O sites with a decreasing solvent polarity is fascinating and impressive.

## 9. Discussion

In Section 4.1 and Section 4.3, we have seen that macrochelate formation involving N7 and the phosphate group of an ANP is more pronounced in the Ni^2+^ complexes than in those containing Cu^2+^. The corresponding observation has been made earlier with Cu(AMP), which has a smaller stability enhancement compared with that of Ni(AMP); of course, the stability enhancement is a reflection of the N7 interaction—i.e., of the extent of macrochelate formation. These observations have been explained by statistical considerations [50]. Assuming Cu has a Jahn–Teller distorted octahedral coordination sphere [157], then there are only the two equatorial *cis* positions of the coordinated phosphate group left, which are able to form macrochelates by coordinating to N7; the *trans* position cannot be reached due to steric restrictions. In the case of Ni^2+^ with its regular octahedral coordination sphere and a bound phosphate group, four *cis* positions for N7 coordination remain; only the *trans* position is again not accessible. Hence, Ni^2+^ compared with Cu^2+^ is statistically favored by a factor of 2 (= 4/2) [50]; i.e., the stability enhancement is expected to be 0.3 log unit larger for Ni(AMP) than Cu(AMP). Indeed, the experimental results (see Equations (12) and (13)) are *Δ* log *Δ*_Ni/Cu/AMP_ = log *Δ*_Ni(AMP)_ − log *Δ*_Cu(AMP)_ = (0.54 ± 0.06) − (0.27 ± 0.08) = 0.27 ± 0.10 (from Column 4 in Table VI of [50]) and thus close to the statistical expectation.

In Section 4.1, we dealt with PME2AP, an ANP isomer in which the amino group is shifted to C2 making for steric reasons N3 less accessible but favoring N7. From the results listed in Table 3, we learned that macrochelate formation with N7 is more pronounced in Ni(PME2AP) (formation degree = % Ni(PME2AP)_cl/N7_ = 85 ± 4%) than in Cu(PME2AP) (73 ± 6%). Application of the above procedure gives *Δ* log *Δ*_Ni/Cu/PME2AP_ = *Δ* log *Δ*_Ni(PME2AP)_ − *Δ* log *Δ*_Cu(PME2AP)_ = (0.82 ± 0.11) − (0.58 ± 0.11) = 0.24 ± 0.16 (data from Column 6 in Table 7 of [43]) indicating the correct trend. The complication is that in the present case not only two isomers (open ⇌ closed N7) form, as with the M(AMP) complexes: Next to these, there is now the five-membered chelate involving the ether oxygen (see structure in Figure 5). To give the complete picture [43], for Ni(PME2AP) = 85 ± 4% (cl/N7), 4 ± 3% (cl/O), and 11 ± 3% (op), and also for Cu(PME2AP) = 73 ± 6% (cl/N7), 18 ± 6% (cl/O), and 9 ± 2% (op).

For PMEDAP^2−^ (structure in Figure 5), the situation is similar, despite the fact that there are two amino groups: one at C2 and one at C6. Due to the increased basicity of the DAP residue [62], the steric hindrance is overcome, and N7 becomes a further binding site leading to macrochelate formation. This then gives rise again to three isomeric complexes: one with the macrochelate, one that involves the ether O atom, plus one open form [62]. The species distribution is as follows: Ni(PMEDAP) = 54 ± 10% (cl/N7), 13 ± 8% (cl/O), and 33 ± 6% (op), and for Cu(PMEDAP) = 43 ± 11% (cl/N7), 38 ± 11% (cl/O), and 19 ± 3% (op) (data from entries 2 and 3 of Table 6 in [62]). The larger formation degree of Ni(PMEDAP)_cl/N7_ confirms also here, despite the large errors, that Ni^2+^ is favored over Cu^2+^.

Above, we have discussed metal ion interactions with N7. In this context one should point out how sensitive these interactions are. For example, solvation of the adenine residue by 1,4-dioxane (in other words, in mixed solvents) inhibits the Cu^2+^–N7 interaction, and this also holds certainly for other metal ions. We assume that the ethylene groups of dioxane undergo hydrophobic interactions with the aromatic residue of the adenine parts and that this screens N7 (Section 8.2). On the other hand, addition of 1,4-dioxane to an aqueous solution reduces the dielectric constant of the solvent, and this favors polar or ionic interactions—e.g., between M^2+^ and a phosph(on)ate group. Such effects (at N and O sites) can give rise to opposing “reactions”, leading thus at a certain solvent mixture to a minimum of the interactions (see Table 11 and Figure 19). Such “solvent effects” can also be thought to be created by amino acid (aa) side chains of proteins. For example, for mixed ligand complexes of the type M(ATP)(aa)^3−^, it is known that the extent of the interaction decreases (in part tentatively) in the series aa = indole residue (tryptophan) > phenyl residue (phenylalanine) ≥ isopropyl residue (leucine) ≥ imidazole residue (histidine) > methyl residue (alanine) (see Section 7.1). Clearly, this can lead to selectivity via the binding strength but also via the orientation in space and therefore, one is in for surprises.

To the best of our knowledge, there is only one study of a larger RNA, where the Mg^2+^ ion binding affinity has been characterized in the presence of 20% 1,4-dioxane [158]. In domain 5 of a group II intron ribozyme, under such conditions, small structural changes were observed in a bulge known to bind Mg^2+^, a new coordination site was detected at a GU wobble pair, and in general the affinity of Mg^2+^ to the RNA increased by a factor of 1.5. More often than the application of 1,4-dioxane to reduce the solvent polarity, other small molecules, sugars as well as polymers such as dextran or PEG (poly-ethylene glycol) have been used to mimic *in cellulo* conditions in in vitro experiments [159]. These substances are called crowding agents, meaning they simulate the tight environment within a cell packed with proteins, biomolecules, and other “spectator” molecules, creating an effect of volume exclusion. However, these molecules also alter the solvent properties, such as the dielectric constant. Generally, such excluded volume effects drastically reduce the requirement for unnaturally high concentration of divalent metal ions needed under traditional in vitro conditions to stabilize the native fold of large RNAs. For example, in a group II intron, the addition of 10% PEG reduces the Mg^2+^ requirement for folding and catalytic activity by a factor of 2 and leads to an increase in catalytic activity by a factor of 4 when keeping the Mg^2+^ concentration constant [160]. Similar effects have been shown for many other classes of ribozymes [159]. In the case of the above-mentioned group II Intron, it was found that the increasing concentration of crowding agent reaches a maximum, after which catalytic activity is again reduced, probably because of too dense crowding becoming detrimental for a correct RNA fold [160].

**Figure 21:**
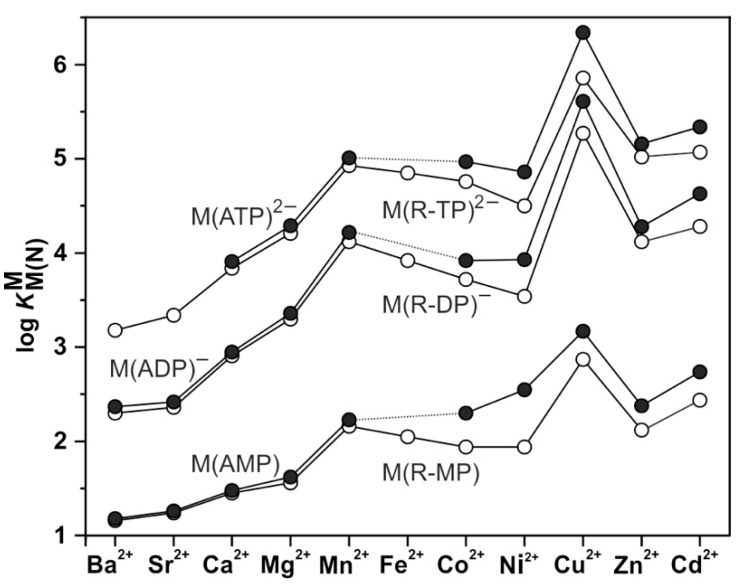
Irving–Williams sequence-type plots [158] for the stability constants log $K_{M(N)}^{M}$ (N = nucleotide) of the 1:1 complexes of Ba^2+^ through Cd^2+^ formed with mono- (R-MP^2−^), di- (R-DP^3−^), and triphosphate monoesters (R-TP^4−^) (◯), as well as of those for AMP^2−^, ADP^3−^, and ATP^4−^ (●). The plotted data of the phosphate ligands are from Table 13 of [23]; they also represent the stability constants for the M^2+^ complexes of the pyrimidine-nucleoside 5′-mono-, di- or triphosphates (except for Cu(CTP)^2−^ [74]). The log stability constants for the M^2+^ complexes of AMP^2−^ and ADP^3−^ are from Table 4 of [153]; those for ATP^4−^ are from Table II of [74] (25 °C; *I* = 0.1 M, NaNO_3_) ([23,74,163,164] as well as pp. 364–365 in [165]).

Another such surprise occurs if one decides to compare the metal ion affinity of the phosphate residues of nucleotides; after all, these phosphate residues are the stability-determining binding sites of the nucleotides. To facilitate this overview, we plotted the logarithmic stability constants log $K_{M(N)}^{M}$, of the mono- (R-MP^2−^), di- (R-DP^3−^), and triphosphates (R-TP^4−^) (R always being a non-interacting residue) in an Irving–Williams-type fashion [161,162] (Figure 21).

Even though known for a long time, it is always a new surprise to observe that plotted data with phosphate ligands do not strictly follow the Irving–Williams sequence [161,162], the Mn^2+^ and the Zn^2+^ complexes are too stable [163,164] (see also pp. 364, 365 in [165]), or one could say that the complexes of the other metal ions are not stable enough; this may be a matter of outersphere complex formation. There is a large stability span of about 1.1 to 2.4 log units, the effect being especially pronounced for Cu^2+^, by going from the M(R-MP) complexes to those of M(R-DP)^−^, whereas the span from M(R-DP)^−^ to M(R-TP)^2−^ amounts only to about 0.8 to 1.0 log unit (if the special case of Cu^2+^ is ignored); this indicates to us that in the M(R-MP) complexes, outersphere binding is of relevance [48,50].

That the phosphate residue plays the dominating part in the determination of complex stability of nucleotides is confirmed by the data that are inserted into Figure 21 for the adenine-nucleotide complexes; these are at least for the third transition metal ions always a touch more stable, which is the result of N7 binding. This small contribution to complex stability should not be misleading; as far as selectivity is concerned, the nucleobases play crucial roles, as we have repeatedly seen. Striking examples for such a selectivity are small ribozymes such as the TS [166] or hammerhead ribozyme: Both classes are most active in the presence of Mn^2+^, with the RzB and *Schistosoma* hammerhead ribozymes experiencing a 400-fold increase in activity over Mg^2+^ [167,168]. Analysis of the cleavage rates in the presence of metal ions along the Irving–Williams series showed a striking parallelism with the metal ion affinity towards the phosphate group [169]. This clearly indicates that the coordination to a phosphate moiety is the decisive factor in positioning the catalytic metal ion with the RNA structure. In contrast, for example in the *glms* ribozyme, named after its cofactor glucosamine-6-phosphate, highest activity is reached in the presence of Mg^2+^, whereas other earth alkaline metal ions and Mn^2+^, Co^2+^, Zn^2+^, and Cd^2+^ inhibit cleavage [170]. Inhibiting effects of divalent metal ions other than Mg^2+^ are also common in the large ribozymes like the group II Intron ribozyme Sc.ai5γ [171]. In such cases, the binding property of the metal ion to other functionals groups, such as the guanine or adenine N7, must play a crucial role too in correctly positioning the metal ion within the RNA structure or even in the catalytic core.

## 10. Conclusions and Outlook

In the preceding part of this review, we have repeatedly seen that metal ions interact with N7 of a purine residue and affect such the structure and function of DNA and RNA molecules [172]. A simple example is the coordination of Pt(II) to N7, which acidifies the (N1)H^+^ site in nucleoside phosphate derivates [173], though to varying extents bio-metals [174] are expected to have the same effect. However, metal ions bind in RNA and DNA not only to N7 but in rare cases also to N1 or N3—e.g., Mg(II) binds in an innersphere to the N3 position of a guanine residue within an adenine riboswitch aptamer (Mg105 to G6-N3) [175,176]. It is therefore of interest to quantify the basicity of the various N sites because commonly complex stability increases with basicity (Section 2). For adenosine (the structure is given in Figure 1), the micro acidity constants have been determined [177]; they are ${pk}_{N7-N1\cdot H}^{N7-N1}$ = 3.63 ± 0.02, ${pk}_{H\cdot N7-N1}^{N7-N1}$ = 2.15 ± 0.15, and ${pk}_{H\cdot N3-N1,N7}^{N3-N1,N7}$ = 1.5 ± 0.3. For example, the value for ${pk}_{H\cdot N7-N1}^{N7-N1}$ quantifies the basicity/acidity of the N7 site under conditions where the N1 site does not carry a proton. Analogously, the value of 1.5 for p*k*_H·N3_ holds for conditions when N1 and N7 are free. Hence, not only for N1 but also for N7 and N3 some metal ion affinity is expected, and this is of relevance for nucleic acids since adenine N7 is exposed in the major and N3 in the minor groove [178,179].

In this review, we have also dealt with acyclic nucleoside phosphonates, which have antiviral properties. An important representative is (*S*)-9-[3-hydroxy-2-(phosphonomethoxy)propyl]adenine (HPMPA), which is active against a range of DNA viruses [180]. The acid–base properties of this compound have recently been studied in detail [181]. The basicity of the adenine nitrogens decreases in the order N1 > N7 > N3, like it was observed for adenosine [177]. The micro acidity constant for ^+^H·N7(HPMPA)N1 was estimated to be ${pk}_{H\cdot N7-N1}^{N7-N1}$ ca 3.5; the acid–base properties of PMEA are expected to be similar [181,182]. The metal ion-coordinating properties of HPMPA have also been studied [183], and it is interesting to compare these with those of (*S*)-1-[3-hydroxy-2-(phosphonomethoxy)propyl]cytosine (HPMPC); note, the cytosine residue does not affect complex stability [184]. It may be added that HPMPC, known as cidofovir [185], is used for the treatment of a variety of DNA virus infections [186]. One may also mention here Acyclovir, which after the addition of a phosphonate group gives the ANP^2−^ = guanine(N9)–CH_2_–O–CH_2_–CH_2_–${\mathrm{PO}}_{3}^{2–}$; one may consider this ANP^2−^ as a relative of PMEA^2−^ in which the adenine residue is replaced by a guanine one and where the "aliphatic" chain is also altered but the number of four chain links is kept. Acyclovir is used to treat herpes infections including outbreaks of genital herpes [187].

Coordination to N7 of purine moieties and associated macrochelate formation is also highly relevant and regularly observed in large nucleic acid structures—i.e., complex RNA molecules such as ribozymes, riboswitches, and other functional RNAs. In such cases, mostly Mg^2+^ is involved, with the Mg^2+^ being party dehydrated, forming innersphere as well as outersphere contacts to the nucleotide moieties. Depending on the local structure, the macrochelate is also formed, not necessarily within a given nucleotide—i.e., from the bridging phosphate to its own nucleobase—but more often between phosphate and N7 of two nucleotides far apart in the primary structure but close in space in the tertiary fold. Prominent examples of such phosphate-purine macrochelate formation by Mg^2+^ are structural metal ion binding motifs such as tandem GC and sheared GA base pairs. Large RNA structures such as the *H. marismortui* large ribosomal subunit [188] contain numerous of such Mg^2+^ binding sites, but they are also known from domains of the self-splicing yeast group II intron Sc.ai5γ [189,190,191], and the stem loop C of a Mg^2+^ riboswitch [192], as is summarized in Ref [163].

An interesting case is the self-activation of Cu_2_(ATP) via dimerization, [Cu_2_(ATP)]_2_ (Figure 10). In this dimer, one half of the complex provides the structure and the other half undergoes the reaction. It is especially fascinating that the structural part of one ATP^4−^ can be taken over by AMP^2−^ or even PMEA^2−^ (Figure 10 and Figure 11). Nonetheless, the smallest structural alteration at the adenine residue (Figure 12) kills the activity. Though apparently simple, the situation is very sensitive, but it corresponds to the M(α,β)-M(γ) binding mode.

Furthermore, nucleoside 5′-triphosphates are in the form of their metal ion complexes important substrates for many reactions of biological relevance. There are two principal structural types—one with the metal ion coordinated as is indicated in the upper part of Figure 2, where the γ-phosphate group is transferred and we observe a kinase-type reaction that in the simplest case is a phosphoryl transfer to water; i.e., the NTP is hydrolyzed. If an NTP is exposed to an excess of metal ions, an M(α,β)-M(γ)-type coordination results. This is different for the structure shown in the middle part of Figure 2, which is of the M(α)-M(β,γ) type and leads to the transfer of a nucleotidyl group—that is, a polymerase-type reaction. In order to achieve this binding mode, the two metal ions need to be anchored by the enzyme; this fixation process can occur via amino acid side chains (such as carboxylate or imidazole groups) of the protein, but fixation via stacking is also possible.

This alternate positioning of two Mg^2+^ along the triphosphate chain is used by two prominent classes of enzymes: In a kinase-type reaction (i.e., the transfer of the γ-phosphate, a typical “phosphoryl-transfer” type of Mg^2+^ coordination is observed), for example, in a phosphoenolpyruvate carboxykinase, the two divalent metal ions (Mn^2+^ and Mg^2+^ in this case) coordinate to the ATP triphosphate chain in a M(α,β,γ)-M(γ)-fashion [24]. In contrast, a polymerase-type coordination is observed in T7 RNA polymerase [193] as well as in a high-fidelity DNA polymerase RB69 [26]. In addition, the employment of two metal ions is also known from ribozymes, where a classical two-metal ion mechanism is used to cleave the RNA chain at a specific phosphodiester—e.g., in group I introns [194,195] and in group II introns [196].

For a metal ion to be shifted along a triphosphate chain into the reactive position, Mg^2+^ appears to be ideal because in the initial 1:1 complex it seems to be present as a mixture of β,γ-bidentate and α,β,γ-tridentate species [197]. Mg^2+^ is by itself not very reactive, but in combination with another metal ion it is quite useful (Figure 8).

## Figures and Tables

**Table 1:** Negative logarithm of the acidity constants (Equations (1) and (2)) for methyl phosphoric acid and methylphosphonic acid [H_2_(P)] in aqueous solution at 25 °C and *I* = 0.1 M (NaNO_3_) *^a^*.

H_2_(P)	${pK}_{H_{2}\left(P\right)}^{H}$	${pK}_{H(P)}^{H}$	Ref *^b^*
CH_3_OPO(OH)_2_	1.1 ± 0.2	6.36 ± 0.01	[40]
CH_3_PO(OH)_2_	2.10 ± 0.03	7.51 ± 0.01	[41]

*^a^* So-called practical, mixed or Brønsted constants [39] are listed—i.e., for the H^+^ ion not the concentration but the activity (as measured by a glass electrode) is used in the calculation of an acidity constant (Equations (1) and (2)). The practical constant may be converted into the concentration constant by subtracting 0.02 from the listed p*K*_a_ values (25 °C; *I* = 0.1 M, NaNO_3_).—The error limits correspond to three times the standard error of the mean value or the sum of the probable systematic errors, whichever is larger. *^b^* See also Refs [42,43].

**Table 2:** Stability enhancements (Equation (12)) for the M(PMEA) and M(PME) complexes and their comparison according to Equation (13). In addition, the extent of chelate formation according to Equilibrium (5) is given as expressed by the dimension-less equilibrium constant *K*_I_ (Equations (6) and (10)) and the percentages of M(PME)_cl_ (Equation (14)). (Aqueous solution; 25 °C; *I* = 0.1 M, NaNO_3_) *^a,b^*.

M^2+^	log *Δ*_M(PMEA)_	log *Δ*_M(PME)_	*Δ* log *Δ*	*K*_I/PME_	% M(PME)_cl_
Mg^2+^	0.16 ± 0.05	0.22 ± 0.03	–0.06 ± 0.06	0.66 ± 0.12	40 ± 4
Ca^2+^	0.11 ± 0.07	0.14 ± 0.05	–0.03 ± 0.09	0.38 ± 0.16	28 ± 9
Sr^2+^	0.07 ± 0.05	0.07 ± 0.05	0.00 ± 0.07	0.17 ± 0.14	15 ± 10
Ba^2+^	0.08 ± 0.06	0.10 ± 0.05	–0.02 ± 0.08	0.26 ± 0.14	21 ± 9
Mn^2+^	0.21 ± 0.08	0.27 ± 0.05	–0.06 ± 0.09	0.86 ± 0.23	46 ± 7
Co^2+^	0.28 ± 0.07	0.29 ± 0.06	–0.01 ± 0.09	0.95 ± 0.28	49 ± 7
Ni^2+^	0.30 ± 0.07	0.19 ± 0.05	0.11 ± 0.09	0.55 ± 0.19	35 ± 8
Cu^2+^	0.77 ± 0.07	0.48 ± 0.07	0.29 ± 0.10	2.02 ± 0.47	67 ± 5
Zn^2+^	0.30 ± 0.10	0.34 ± 0.06	–0.04 ± 0.12	1.19 ± 0.32	54 ± 7
Cd^2+^	0.33 ± 0.05	0.30 ± 0.05	0.03 ± 0.08	1.00 ± 0.25	50 ± 6

*^a^* The values are compiled from Tables 2 and 3 of [45]. For the error limits, see the terminal sentence of Footnote ‘*a*’ in Table 1; derived data were calculated according to the error propagation after Gauss. *^b^* Acidity constants: ${pK}_{H_{2}\left(\mathrm{PMEA}\right)}^{H}$ = 4.16 ± 0.02 (deprotonation of the (N1)H^+^ site), ${pK}_{H(\mathrm{PMEA})}^{H}$ = 6.90 ± 0.01 and ${pK}_{H(\mathrm{PME})}^{H}$ = 7.02 ± 0.01 (deprotonation of the P(O)_2_(OH) residue) (from Table 2 in [45]).

**Table 3:** Formation degrees of the isomeric complexes, M(PME2AP)_op_, M(PME2AP)_cl/O_, and M(PME2AP)_cl/N7_ (Equilibrium Scheme (15) in Figure 6), in aqueous solution at 25 °C and *I* = 0.1 M (NaNO_3_) *^a^*.

M^2+^	% M(PME2AP)_op_	% M(PME2AP)_cl/O_	% M(PME2AP)_cl/N7_
Mg^2+^	63 ± 7	37 ± 7	~0
Ca^2+^	74 ± 14	26 ± 14	~0
Mn^2+^	59 ± 8	41 ± 8	~0
Co^2+^	24 ± 4	14 ± 6	62 ± 7
Ni^2+^	11 ± 2	4 ± 3	85 ± 4
Cu^2+^	9 ± 2	18 ± 6	73 ± 6
Zn^2+^	13 ± 3	12 ± 5	75 ± 6
Cd^2+^	35 ± 7	35 ± 11	30 ± 13

*^a^* Regarding the error limits (3σ) see Footnote ‘*a*’ in Table 2. This table is based with permission on our publication in *Dalton Transactions* [43], copyright 2010, The Royal Society of Chemistry.

**Table 4:** Stability enhancements (Equation (12)) for the M(PMEDAP) and M(PME-R) complexes and their comparison according to Equation (13). In addition, the extent of chelate formation according to Equilibrium (5) is given as expressed by the dimension-less equilibrium constant *K*_I_ (Equations (6) and (10)) and the percentages of M(PMEDAP)_cl/O_ (Equation (14)). (Aqueous solution; 25 °C; *I* = 0.1 M, NaNO_3_) *^a^*.

M^2+^	log *Δ*_M(PMEDAP)_	log *Δ*_M(PME-R)_	*Δ* log *Δ*	*K*_I/PMEDAP_	%M(PMEDAP)_cl/O_
Mg^2+^	0.17 ± 0.05	0.16 ± 0.04	0.01 ± 0.06	0.48 ± 0.17	32 ± 8
Ca^2+^	0.12 ± 0.06	0.12 ± 0.05	0.00 ± 0.08	0.32 ± 0.19	24 ± 11
Sr^2+^	0.08 ± 0.04	0.09 ± 0.05	–0.01 ± 0.06	0.20 ± 0.12	17 ± 9
Ba^2+^	0.10 ± 0.06	0.11 ± 0.05	–0.01 ± 0.08	0.26 ± 0.19	21 ± 12
Mn^2+^	0.18 ± 0.06	0.19 ± 0.06	–0.01 ± 0.08	0.51 ± 0.20	34 ± 9
Co^2+^	0.33 ± 0.07	0.20 ± 0.06	(0.13 ± 0.09) *^b^*	(1.14 ± 0.36)*^c^*	(53 ± 8) *^c^*
Ni^2+^	0.48 ± 0.08	0.14 ± 0.07	(0.34 ± 0.11) *^b^*	(2.02 ± 0.54)*^c^*	(67 ± 6) *^c^*
Cu^2+^	0.73 ± 0.07	0.48 ± 0.07	(0.25 ± 0.10) *^b^*	(4.37 ± 0.89) *^c^*	(81 ± 3) *^c^*
Zn^2+^	0.40 ± 0.11*^d^*	0.29 ± 0.07	0.11 ± 0.13	1.51 ± 0.63	60 ± 10 *^d^*
Cd^2+^	0.32 ± 0.13	0.30 ± 0.05	0.02 ± 0.14	1.09 ± 0.63	52 ± 14

*^a^* The values are compiled from Tables 4 and 5 of [62]. For the error limits (3δ), see Footnote ‘*a*’ in Table 2. *^b^* These values are larger than zero, indicating that also a metal ion–nucleobase interaction occurs (see text). *^c^* Because *Δ* log *Δ* > 0, the *K*_I_ and the % M(PMEDAP)_cl/O_ values also contain a contribution of the M(PMEDAP)_cl/N_ species. *^d^* The stability of the Zn(PMEDAP) complex contains probably also a contribution from a nucleobase–Zn^2+^ interaction (for details see Table 6 and Footnote ‘*b*’ of Table 6 in [62]).

**Table 5:** Stability enhancements (Equation (12)) for the M(PME2A6DMAP) and M(PME-R) complexes and their comparison according to Equation (13). In addition, the extent of chelate formation according to Equilibrium (5) is given as expressed by the dimension-less equilibrium constant *K*_I_ (Equations (6) and (10)) and the percentages of M(PME2A6DMAP)_cl/O_ (Equation (14)). The values for % M(PME-R)_cl/O_ are given for comparison. (Aqueous solution; 25 °C; *I* = 0.1 M, NaNO_3_) *^a^*.

PE^2−^ = PME2A6DMAP^2−^						
M^2+^	Log *Δ*_M(PE)_	log *Δ*_M(PME-R)_	*Δ* log *Δ*	*K*_I/PE_	% M(PE)_cl/O_	% M(PME-R)_cl/O_
Mg^2+^	0.16 ± 0.04	0.16 ± 0.04	0.00 ± 0.06	0.45 ± 0.13	31 ± 6	31 ± 6
Ca^2+^	0.10 ± 0.05	0.12 ± 0.05	−0.02 ± 0.07	0.26 ± 0.14	21 ± 9	24 ± 9
Sr^2+^	0.11 ± 0.05	0.09 ± 0.05	0.02 ± 0.07	0.29 ± 0.15	22 ± 9	19 ± 9
Ba^2+^	0.08 ± 0.06	0.11 ± 0.05	−0.03 ± 0.08	0.20 ± 0.17	17 ± 11	22 ± 9
Mn^2+^	0.19 ± 0.06	0.19 ± 0.06	0.00 ± 0.08	0.55 ± 0.21	35 ± 9	35 ± 9
Co^2+^	0.21 ± 0.07	0.20 ± 0.06	0.01 ± 0.09	0.62 ± 0.26	38 ± 10	37 ± 9
Ni^2+^	0.17 ± 0.07	0.14 ± 0.07	0.03 ± 0.10	0.48 ± 0.24	32 ± 11	28 ± 12
Cu^2+^	0.56 ± 0.07	0.48 ± 0.07	0.08 ± 0.10	2.63 ± 0.59	72 ± 4	67 ± 5
Zn^2+^	0.28 ± 0.07	0.29 ± 0.07	−0.01 ± 0.10	0.91 ± 0.31	48 ± 8	49 ± 8
Cd^2+^	0.25 ± 0.06	0.30 ± 0.05	−0.05 ± 0.08	0.78 ± 0.25	44 ± 8	50 ± 6

*^a^* The values are compiled from Tables 4 and 5 of [61]. For the error limits (3σ), see Footnote ‘*a*’ in Table 2.

**Table 6:** Equilibrium constants defined according to the isodesmic model for a non-cooperative self-association for the self-stacking (Equations (18) and (19)) of adenosine and its 5′-phosphates as well as of M(ATP)^2−^ complexes, as determined by ^1^H NMR shift measurements [90,94] in D_2_O (27 °C; *I* = 0.1 − ~2 M NaNO_3_) (error limits: 2σ).

No.	System	*K* (M^−1^)
1	Ado	15 ± 2
2	AMP^2−^	2.1 ± 0.4
3	ADP^3−^	1.8 ± 0.5
4	ATP^4−^	1.3 ± 0.2
5	Mg(ATP)^2−^	4.0 ± 0.5
6	Zn(ATP)^2−^	~11.1 ± 4.5
7	Cd(ATP)^2−^	~17

Reproduced with permission from our publication in *Pure and Applied Chemistry* [21]; copyright 2004, International Union of Pure and Applied Chemistry (IUPAC).

**Table 8:** Intramolecular equilibrium constants for the formation of the three differently structured Cu(arm)(PMEA) species shown in the Equilibrium Scheme (24), together with the percentages in which these species occur in aqueous solution at 25 °C and *I* = 0.1 M (NaNO_3_) *^a^*.

arm	*K*_I_ = *K*_I/tot_ (Equation (29))	% Cu(arm)(PMEA)_int/tot_ (cf. Equation (29))	% Cu(arm)(PMEA)_op_ (Equation (24))	*K*_I/O_ (Equation (27))	*K*_I/st_ (Equations (27) and (29)) *^c^*	% Cu(arm)(PMEA)_cl/O_ (Equations (5) and (24)) *^b^*	% Cu(arm)(PMEA)_st_ (Equation (24)) *^c^*
bpy	29.20 ± 4.87	96.69 ± 0.53	3.31 ± 0.53	2.89 ± 0.68	26.31 ± 4.92	10 ± 3	87 ± 3 (21)
phen	53.95 ± 8.86	98.18 ± 0.29	1.82 ± 0.29	3.17 ± 0.69	50.78 ± 8.89	6 ± 2	92 ± 2 (25)

*^a^* The values listed in the second column are from the fifth column in the lower part of Table 7. These values for *K*_I_ = *K*_I/tot_ follow from Equation (29), and % Cu(arm)(PMEA)_int/tot_ is calculated analogously to Equation (14). The values given in the fourth column for % Cu(arm)(PMEA)_op_ follow from 100 − % Cu(arm)(PMEA)_int/tot_. The constants *K*_I/O_ of Column 5 are from the upper part in Column 5 of Table 7 (for the corresponding justification see text in Section 7.2); with Equation (26) and the now known values for *K*_I_ and *K*_I/tot_ that for *K*_I/st_ may be calculated (Column 6). All error limits in this table correspond to three times the standard deviation (3σ); they were calculated according to the error propagation after Gauss. *^b^* These values were calculated via Equation (26) with *K*_I/O_ and % Cu(arm)(PMEA)_op_. *^c^* The values for % Cu(arm)(PMEA)_st_ follow from the difference % Cu(arm)(PMEA)_int/tot_ − % Cu(arm)(PMEA)_cl/O_ (cf. Equation (29)); % Cu(arm)(PMEA)_st_ may also be calculated via Equation (27) with *K*_I/st_ and % Cu(arm)(PMEA)_op_. The results are the same for both calculation methods (aside from the possible differences in the last digit due to differences in rounding), yet the error limits (which are given in parenthesis) are understandably larger for the second method. This table is reproduced (slight alterations were made) with permission from Table 9 in our publication in *Coordination Chemistry Reviews* [45]; copyright 1995, Elsevier Science S.A., Lausanne, Switzerland.

**Table 9:** Extent of chelate formation according to Equilibrium (5) in the Cu(PME) species as quantified by the dimension-less equilibrium constant *K*_I_ (Equations (6) and (10)) and the percentage of Cu(PME)_cl_ (Equation (14)) in aqueous solution and in water containing 30 or 50% (*v*/*v*) 1,4-dioxane together with properties of the mentioned solvents (25 °C; *I* = 0.1 M, NaNO_3_)*^a^*.

No.	% Dioxane (*v*/*v*)	mol Fraction	ε *^b^*	log *Δ*_Cu(PME)_ Equation (12)	*K*_I_ Equations (6) and (10)	% Cu(PME)_cl_ Equation (14)
1	0	0	78.5	0.48 ± 0.07	2.02 ± 0.47	67 ± 5
2	30	0.083	52.7	0.39 ± 0.04	1.45 ± 0.24	59 ± 4
3	50	0.175	35.2	0.41 ± 0.07	1.57 ± 0.40	61 ± 6

*^a^* The values for log *Δ*_Cu(PME)_ (see also Figure 18), *K*_I_, and % Cu(PME)_cl_ are from Table 5 of [149] (see also Table 6 of [45]). The information about the solvents is from Table 2 of [149] (see also Table 5 of [45]). For the error limits (3σ) see Footnote ‘*a*’ in Table 2. *^b^* The dielectric constants for the dioxane-water mixtures are interpolated from the data given in [147,148] (see also [45,149]).

**Table 10:** Effect of 1,4-dioxane (*v/v*) on the stability of stacks formed in various systems and various solvents *^a^*.

		Solvent			
No.	System	Water	30% Diox. *^a^*	50% Diox. *^a^*	Comments
(1.a)	phen (self.)	31.1 ± 3.4	2.63 ± 0.44	0.63 ± 0.13	^1^H NMR; D_2_O *^b,c^*
(1.b)	bpy (self.)	7.4 ± 2.3	0.86 ± 0.21	0.38 ± 0.06	^1^H NMR; D_2_O *^b,c^*
(2.a)	(phen)(ATP)^4−^	26.8 ± 7.4 (38)	4.1 ± 1.1 (4.8)	1.6 ± 0.7 (1.8)	^1^H NMR; D_2_O *^c,d^*
(2.b)	(bpy)(ATP)^4−^	13.6 ± 3.9 (16)	3 ± 2 (3.5)	0.3 ± 0.2 (0.4)	^1^H NMR; D_2_O *^c,d^*
(3.a)	Cu(phen)(ATP)^2−^	0.56 ± 0.08	–0.03 ± 0.05	–0.41 ± 0.09	Pot. *^e,f^*
(3.b)	Cu(bpy)(ATP)^2−^	0.33 ± 0.04	–0.14 ± 0.05	–0.46 ± 0.07	Pot. *^e,f^*
(4.a)	Cu(UTP)^2−^	5.81 ± 0.06	6.16 ± 0.05	6.24 ± 0.03	Pot. *^f,g^*
(4.b)	Cu(ATP)^2−^	6.32 ± 0.04	6.40 ± 0.05	6.34 ± 0.05	Pot. *^f,g^*

*^a^* The values were collected from Tables 1, 2, and 4 of Ref [127]. For the properties of the dioxane-water mixtures, see Table 9. In the NMR experiments, deuterated dioxane was used. *^b^* $K_{\mathrm{arm}}^{\mathrm{self}}$ (self-association constant according to the isodesmic model; see Equations (18) and (19)). *^c^* The error limits correspond to twice the standard deviation (2σ). 27 °C; *I* = 0.1 M (NaNO_3_). *^d^* Stability constant $K_{\left(\mathrm{arm})(\mathrm{ATP}\right)}^{\mathrm{ATP}}$ for the (arm)(ATP)^4−^ adduct; the values in parentheses are corrected for the self-association of arm (= phen or bpy). *^e^* Listed are the values for *Δ* log *K*_Cu/arm/ATP_ (Equation (30)) which quantify Equilibrium (31). *^f^* Values based on potentiometric pH titrations [127]. The errors given are three times the standard error of the mean value (3σ); where needed the error propagation after Gauss was employed. 25 °C; *I* = 0.1 M, NaNO_3_. *^g^* Listed are the log stability constants for the binary Cu(NTP)^2−^ complexes—that is, log $K_{\mathrm{Cu}(\mathrm{NTP})}^{\mathrm{Cu}}$ [127].

**Table 11:** Extent of chelate formation involving Cu^2+^ and the adenine residue (AD) of AMP^2−^ and PMEA^2−^ (analogous to Equation (14)) in aqueous solution and in water containing varying percentages (*v*/*v*) of 1,4-dioxane at 25 °C and *I* = 0.1 M (NaNO_3_). The alterations are quantified via log *Δ*_Cu(AMP)_ and compared with log *Δ*_Cu(PMEA)_ (Equation (12)). These data are based on the stability of the Cu(AMP) and Cu(PMEA) complexes (analogous to Equation (8)) *^a,b^*.

AD*^c^*	% (*v*/*v*) Dioxane	**log** $\;K_{\mathrm{Cu}(\mathrm{AD})}^{\mathrm{Cu}}$	**log** $\;K_{{\mathrm{Cu}(\mathrm{AD})}_{\mathrm{op}}}^{\mathrm{Cu}}$	log *Δ*_Cu(AD)_	*K*_I_	% Cu(AD)_cl_
AMP^2−^	0	3.14 ± 0.01	2.87 ± 0.08	0.27 ± 0.08	0.86 ± 0.35	46 ± 10
	20	3.56 ± 0.01	3.49 ± 0.04	0.07 ± 0.04	0.17 ± 0.11	15 ± 8
	30	3.86 ± 0.02	3.82 ± 0.03	0.04 ± 0.04	0.10 ± 0.09	9 ± 8
	40	4.30 ± 0.02	4.19 ± 0.06	0.11 ± 0.06	0.29 ± 0.19	22 ± 11
	50	4.73 ± 0.04	4.45 ± 0.02	0.28 ± 0.04	0.91 ± 0.20	48 ± 5
PMEA^2−^	0	3.96 ± 0.04	3.19 ± 0.06	0.77 ± 0.07	4.89 ± 0.98	83 ± 3 (49 ± 10) *^c^*
	30	4.70 ± 0.05	4.18 ± 0.03	0.52 ± 0.06	2.31 ± 0.44	70 ± 4 (26 ± 10) *^c^*
	50	5.54 ± 0.07	4.79 ± 0.03	0.75 ± 0.08	4.62 ± 0.99	82 ± 3 (54 ± 9) *^c^*

*^a^* The data for Cu(AMP) are from the Table in [146] and those for Cu(PMEA) from Tables 5 and 6 of [45] (for Cu(AMP) see also [149]). For further details (error limits, etc.), see Footnote ‘*f*’ of Table 10. *^b^* Relevant definitions are given in Equation (6) through Equations (12) and (14), where PE^2−^ = AMP^2−^ or PMEA^2−^. *^c^* AMP^2−^ forms in Cu(AMP) macrochelates with N7 (see, e.g., [21,46,50,53,95,153]). PMEA^2−^ may form several chelates in M(PMEA) complexes (see Section 4.2); we consider here the total amounts of chelates formed, Cu(PMEA)_cl/tot_, but it may be assumed that Cu(PMEA)_cl/O/N3_ dominates (Section 3 and Section 4.2). For this reason, the calculated values for Cu(PMEA)_cl/O/N3_ listed in Table 7 of [45] are also given in parentheses; the small concentrations of Cu(PMEA)_cl/N7_ are ignored in the above numbers.

## Abbreviations and Definitions

aa	amino acid
AcP^2−^	acetyl phosphate (Figure 20)
AIDS	acquired immune deficiency syndrome
AMP·NO^2−^	adenosine 5′-monophosphate N(1)-oxide (Figure 12)
AMP^2−^	adenosine 5′-monophosphate (Figure 3)
ε-AMP^2−^	1,*N*^6^-ethenoadenosine 5′-monophosphate (Figure 12)
AnP^2−^	acetonylphosphonate (Figure 20)
arm	heteroaromatic nitrogen base, e.g., bpy or phen
ATP^4−^	adenosine 5′-triphosphate
bpy	2,2-bipyridine
DHAP^2−^	dihydroxyacetone phosphate (Figure 20)
dien	diethylenetriamine = 1,4,7-triazaheptane
DNA	deoxyribonucleic acid
dPMEA^2−^	dianion of 9-(4-phosphonobutyl)adenine = 3′-deoxa-PMEA^2−^
G1P^2−^	glycerol 1-phosphate (= α-glycerophosphate; in many biochemistry texts also designated as glycerol 3-phosphate) (Figure 20)
HIV	human immunodeficiency virus
L^2−^	any phosph(on)ate ligand (R-${\mathrm{PO}}_{3}^{2–}$)
M^2+^	any divalent metal ion (in a few instances also Cu(bpy)^2+^ and Cu(phen)^2+^ are represented by this abbreviation)
N	nucleophile
NDP^3−^	nucleoside 5′-diphosphate
NMP^2−^	nucleoside 5′-monophosphate
NTP^4−^	nucleoside 5′-triphosphate
PEEA^2−^	dianion of 9-[2-(2-phosphonoethoxy)ethyl]adenine
phen	1,10-phenanthroline
PME^2−^	dianion of (phosphonomethoxy)ethane (=ethoxymethanephosphonate) [Equation (5)]
PMEA^2−^	dianion of 9-[2-(phosphonomethoxy)ethyl]adenine (Figure 3)
RNA	ribonucleic acid
TuMP^2−^	tubercidin 5′-monophosphate = 7-deaza-AMP^2−^ (Figure 12)

Several additional definitions are given in the legends to Figure 1, Figure 2, Figure 4 and Figure 5.—Species that are given in the text without a charge either do not carry one or represent the species in general (i.e., independent of their degree of protonation); which of the two versions applies is always clear from the context.
